# Comparative Physiological and Transcriptomics Profiling Provides Integrated Insight into Melatonin Mediated Salt and Copper Stress Tolerance in *Selenicereus undatus* L.

**DOI:** 10.3390/plants13243602

**Published:** 2024-12-23

**Authors:** Darya Khan, Xin Yang, Gong He, Raja Asad Ali Khan, Babar Usman, Liu Hui, Aamir Ali Khokhar, Qamar U Zaman, Hua-Feng Wang

**Affiliations:** 1 Sanya Nanfan Research Institute of Hainan University, Hainan Yazhou Bay Seed Laboratory, Sanya 572025, China; 2 Collaborative Innovation Center of Nanfan and High-Efficiency Tropical Agriculture, School of Tropical Agriculture and Forestry, Hainan University, Haikou 570228, China; 3 College of Tropical Agriculture and Forestry, Hainan University, Haikou 570228, China

**Keywords:** *Selenicereus undatus* L., abiotic stresses, physiology, transcriptomic, WGCNA

## Abstract

*Selenicereus undatus* L., (pitaya) is an important tropical fruit crop, and faces significant challenges from soil salinity and heavy metal toxicity. This study explores the role of melatonin (M) in enhancing stress tolerance in pitaya against salinity (S) and copper (Cu) toxicity, both individually and in combination (SCu). SCu stress reduced plant biomass by ~54%, while melatonin application mitigated stress effects and increased plant growth by ~73.26% under SCuM compared to SCu treatment. Antioxidant activities were also modulated by stress. Transcriptomic analysis revealed 21 differentially expressed genes (DEGs) common across stress treatments and 13 DEGs specific to combined melatonin with stress treatments involved in stress signaling, secondary metabolite biosynthesis, and photosynthesis. A weighted gene co-expression network analysis (WGCNA) identified four gene modules (brown, dark green, dark grey, and grey) significantly associated with phenotypic traits. A protein–protein interaction (PPI) network analysis highlighted 14 hub genes per module, including *GH3, JAZ, PAL, CCR,* and *POD*, implicated in MAPK signaling, phenylpropanoid biosynthesis, and hormone signaling pathways. Integration of DESeq2 and WGCNA identified 12 key stress-responsive genes strongly correlated with phenotypic traits. This study provides insights into regulatory mechanisms underlying stress responses and highlights candidate genes for developing stress-resilient *S. undatus* through breeding programs.

## 1. Introduction

*Selenicereus undatus* L. is a diploid (2n = 2x = 22) vine fruit crop of the family Cactaceae, generally referred as dragon fruit or pitaya [1]. Pitaya is native to Latin America (Mexico and Colombia) and distributed from tropical to subtropical regions worldwide [2,3]. The fruit’s striking appearance, delectable taste, high nutrition, antioxidant capacity, and antiproliferative activities increased its popularity for high cultivation and production [4,5]. As a cactus, pitaya is believed to have a high tolerance to climate change and severe environments, especially variations in CO_2_ levels, high temperature, and drought [6,7]. However, multiple stress factors are influencing crop development and productivity in tropical and subtropical agricultural lands [8,9]. Abiotic stress factors, including prolonged drought, high soil salinity, soil pH level, heavy metal toxicity, extreme temperature, and floods, are the most common factors that reduce crop production by ~50% as compared to biotic factors [10,11]. Among these, soil toxicity due to high concentrations of salt and heavy metals are among the major threats to plant development and production [12,13].

Generally, soil salinity is associated with the presence of sodium (Na^+^), calcium (Ca^2+^), magnesium (Mg^2+^) cations and chlorine (Cl^−^), sulphate (SO_4_^2−^), hydrocarbon (HCO_3−_) anions in soil. It has been reported that approximately 20% of the cultivated lands and 33% of irrigated agricultural lands are highly affected by salinity [14,15]. Salt, in the form of Na^+^Cl^−^, is considered to be the most common cause of salt toxicity in plants [16]. It has been reported that high salt concentrations (~40 mM NaCl or above) cause ionic, osmotic, and oxidative stress in plants which severely affects plant growth, production and biomass by membrane disorganization, production of reactive oxygen species (ROS), thereby causing metabolic toxicity, effect photosynthesis, nutrient uptake, decrease cell division, and accelerate cell death [15,17,18]. Heavy metals, such as copper (Cu), iron (Fe), molybdenum (Mo), zinc (Zn), nickel (Ni), manganese (Mn), and cobalt (Co), in a medium range are essential micronutrients for plant growth and developmental processes, but their high accumulation can cause toxic effects [19,20]. Among these heavy metals, copper (Cu^2+^ and Cu^+^) in high concentrations (<0.20 mM) inhibits plant growth by disrupting multiple plant cellular processes [21,22]. High copper accumulation in plants is considered more toxic for the plant photosynthesis process, as compared to other heavy metals i.e., Cd, Zn, Ni and Pb [23]. An excessive accumulation of copper in the roots and leaves causes oxidative stress which targets almost all cellular macromolecules including lipids, protein, enzymes, DNA, and RNA [24,25].

Under stress conditions, plants integrate signals to activate their defense system and respond morphologically, physiologically, and biochemically [24,26,27] to mitigate osmotic, ionic, and oxidative stresses [25,28,29]. Phytohormones, such as indole-3-acetic acid or auxin (IAA), cytokinin (CK), ethylene (ET), abscisic acid (ABA), gibberellin (GA), brassinosteroid (BR), salicylic acid (SA), jasmonic acid (JA), and strigolactone (SL), are chemical messengers associated with signals transduction as well as ROS and redox signaling; calcium-based signals activate the defense system which help the plant to survive under stress [30,31,32]. Plants have different defense strategies to specific stress; for example, in response to sodium and copper ion toxicity, plants limit their absorption through their roots by selective intake of ions or precipitating the toxic ions in less sensitive tissues. In response to salinity, the roots select potassium (K^+^) ions over sodium (Na^+^) ions and pump out the excess Na^+^ from their roots to maintain a K^+^/Na^+^ ratio for normal cell function [33,34]. Whereas under copper (Cu^+^ and Cu^2+^) stress, the roots release citrate, viz proline, organic acids, and histidine which form an extracellular complex with copper ions and prevent their absorption into root cells. Plants also close their stomata to minimize the intake and translocation of toxic ions into their tissues via transpiration [35,36]. Metabolites, such as proline, glycine betaine, polyamines, sugar, alcohol, and organic acids, have osmolyte functions which help the plant in osmoregulation under drought, cold, and salinity stresses [37,38]. To reduce oxidative stress, the defense system activates enzymatic antioxidants i.e., superoxide dismutases (SODs), catalases (CATs), ascorbate peroxidases (APXs), glutathione peroxidase (GPXs), and non-enzymatic antioxidants i.e., ascorbic acid (vitamin C), α-tocopherols, glutathione (GSH), 3-flavonoids, and carotenoids, which help in scavenging the free radicals and protect the plants from oxidative damage [39,40,41].

Some exogenous applications of growth regulating hormones also help to mitigate stress effects and promote plant growth, i.e., cytokinin, auxin, brassinosteroid, melatonin, and salicylic acid, etc. [42,43,44]. Among these, melatonin was first discovered in plants in 1995 [45,46]. Melatonin occurs naturally and is a non-toxic, multifunctional bio-stimulator, promoting plant growth against both biotic and abiotic environmental stress factors [47,48]. It has been reported that the exogenous application of melatonin enhances the photosynthetic capacity in *Malus hupehensis* [49] and increases root density in *Cucumis sativus* under salt stress [50]. Other studies have reported that exogenous melatonin alleviates copper toxicity in *Cucumis sativus* and *Brassica napus* by scavenging the reactive oxygen species [50,51].

These complex defense processes are regulated by the number of genes, and transcriptional factors belong to various gene families. In rice, the *bHLH* family gene (*OsBHLH148*) regulates jasmonate-signaling pathways [52]; while the *Chrysanthemum WRKY* gene (*CmWRKY10*) regulates ABA-signaling pathways in response to drought [53]. The tomato *BZR/BES* transcription factor (*SIBZR1*) regulates BR signaling in tomatoes; and Arabidopsis [54], rice *SERF1* interact with *MAP3K6*, *MAPK5* to activate MAPK pathways [55]; and *SOS* genes (*SOS1/2&3*) signaling activates SOS pathway in response to salt stress [56]. Similarly, *HMA* genes [57], *MATE* genes [58], and *WRKY* transcription factors [59] regulate a heavy metal stress response in plants. However, the regulation of genes depends on stress type, plant species, and varieties which occurs mostly at transcriptional, post-transcriptional, and post-translation levels. The most important level is the transcriptional level. At the transcriptional level, chromatin remodeling and differential gene expression (DEG) help the plant cell to re-establish cell homeostasis and normal cell function under stress conditions [59,60,61]. RNA sequencing has facilitated the understanding of the molecular mechanism of the plant defense process and the role of stress responsive genes and pathways in stress tolerance, and has been widely studied in many plants species, including Arabidopsis [62], cotton [63], and maize [64] among others. But it is still unknown how pitaya responds to environmental abiotic stress conditions, especially salt and copper toxicity.

This study aims to explore the physiological and molecular mechanisms of pitaya responses towards salt and copper heavy metal toxicity and the effect of exogenous melatonin application as a defensive hormone against salinity and heavy metal stress, especially copper. Our results explore the unique physiological and genetic response of pitaya to various stress treatments and melatonin applications that sufficiently enhanced pitaya growth under stress conditions by regulating stress response pathways.

## 2. Results and Discussion

### 2.1. Plant Growth and Response of Pitaya Towards Single and Combined Treatments

**Figure 1:**
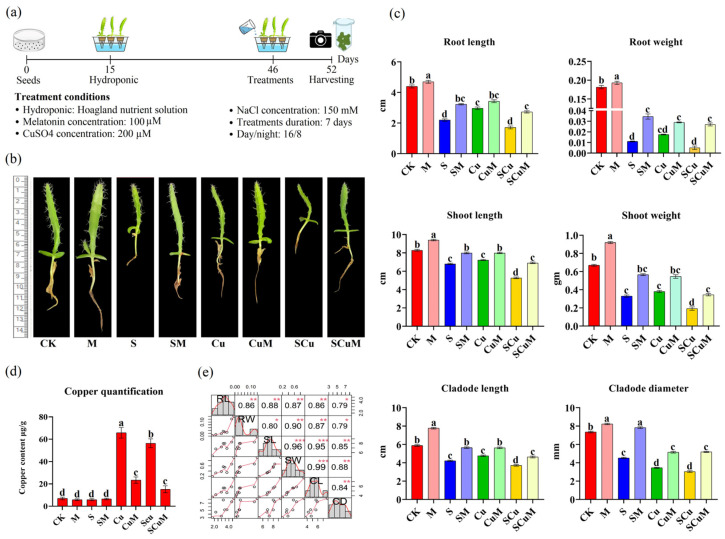
Influence of single and combined stresses treatments of melatonin (M), salt (S), and copper (Cu) on the growth of pitaya seedlings. (**a**) Overview of the experimental steps and treatment conditions followed for the experiment. Plants were harvested and photographed on day 52. (**b**) Phenotype of seedlings on day 52, treated with a total of 8 different treatment combinations [control (CK), M, S, SM, Cu, CuM, SCu, and SCuM]. (**c**) Phenotypic attributes of the seedlings were measured as RL, SL, CL (unit = cm), RW, SW (unit = gm), and CD (unit = mm). According to the Duncan test, different letters indicate a significant difference (*p* < 0.05) among the treatments. (**d**) Quantification of Cu heavy metal (unit = μg/g) in cladode tissue under single and combined abiotic stress treatments. (**e**) Significant phenotypic correlation between the root length (RL), root weight (RW), cladode length (CL), cladode diameter (CD), shoot length (SL), and shoot weight (SW). Significant and highly significant correlations are represented by * (*p* < 0.05), ** (*p* < 0.01) and *** (*p* < 0.001). The error bars represent the mean ± SE.

Among the multiple abiotic stresses, salt (S) and copper (Cu) toxicity both have adverse effects on plant growth and morphology, including plant shoot length, root length, and plant biomass [65]. These stresses cause chlorosis, necrosis, stunting, senescence, dehydration, and softening of tissues [66,67,68]. Melatonin (M) is a growth regulating hormone, studied widely in several plants, which promotes plant growth and alleviates stress effects [69,70]. To study the adverse effects of S and Cu toxicity in pitaya and the role of M in abiotic stress tolerance, we sowed pitaya seeds in petri plates and then transferred them to hydroponic pots containing Hoagland nutrient solution [71]. We applied single and combined treatments of M, S, and Cu for one week to the 45-day-old seedlings with a cladode length of ~3 cm (Figure 1a). The treatments were applied as control (CK), M, S, SM, Cu, CuM, SCu, and SCuM. The results showed obvious differences in seedling morphology under single and combined treatments (Figure 1b). The seedling parameters for CK were recorded as follows: RL (4.39 cm), RW (0.18 g), SW (8.29 cm), SW (0.66 g), CL (5.88 cm), and CD (7.35 mm). Exogenous application of M enhanced the plant growth with ~16.6% increase, as compared to CK. The S treatment significantly affected the seedlings growth by ~42.50% decrease, as compared to CK. Symptoms of dehydration, softening of tissues, and necrosis were observed in the S treated samples. In Cu treated samples chlorosis, necrosis, and stunting of tissues were observed and ~41.31% decrease was recorded in seedling growth, as compared to CK. Whereas, the combined stress treatment of S and Cu (SCu) extensively affected the seedlings growth with ~54% decrease, as compared to CK. Severe symptoms of chlorosis, necrosis, stunting, senescence, dehydration, and softening of tissues were observed. The M application in combination with S (SM), Cu (CuM), and SCu (SCuM) were applied to the seedlings. The results showed an increase in the seedling growth, treated with SM (~58.03%), CuM (~47.87%), and SCuM (~73.26%), as compared to S, Cu, and SCu, respectively. Severe symptoms were reduced in each stress-treated sample in combination with the M treatment (Figure 1b,c). Quantification of Cu heavy metal in the cladode tissue was conducted for each treatment sample. The quantification results showed the highest accumulation of Cu heavy metal in the samples treated with Cu (~68.8 μg/g), followed by the SCu (~56.39 μg/g) treated sample. The M application sufficiently decreased Cu accumulation in both CuM (65.94%) and SCuM (73.13%) treated samples, as compared to Cu and SCu, respectively (Figure 1d). The relationship between phenotypes showed a highly significant and positive correlation among SL/SW/CD (*p* < 0.001), CD/CL/SW/SL (*p* < 0.01), RL/RW/SL/SW/CL (*p* < 0.001), and was significant among RW/CD and RL/CD (*p* < 0.05) (Figure 1e). These results were compared with previous studies of *Solanum lycopersicum* [72], *Gossypium hirsutum* [73], and *Cucumis sativus* [74] revealing the toxic effect of salt and copper stresses on plant growth and development; whereas, melatonin promoted plant growth, alleviated stress effects, and enhanced tolerance against multiple abiotic stresses.

### 2.2. Antioxidative Response of Pitaya Towards Single and Combined Treatments

**Figure 2:**
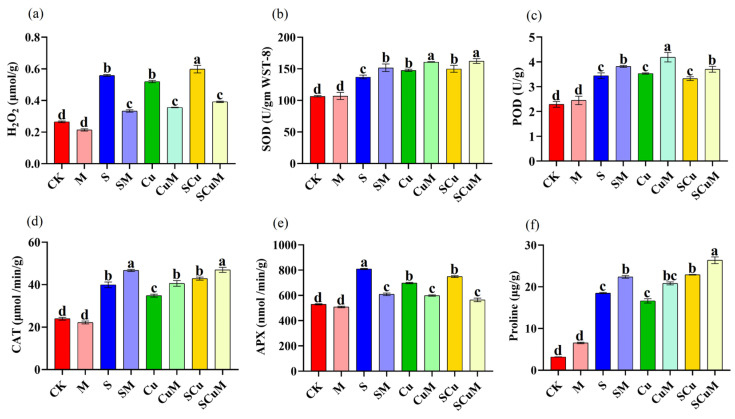
Antioxidative response of pitaya towards single and combined abiotic stress treatments including control (CK), melatonin (M), salt (S), and copper (Cu). (**a**) Generation of ROS (H_2_O_2_) under all treatments, (**b**) Superoxide dismutase (SOD), (**c**) Peroxidase (POD), (**d**) Catalase (CAT), (**e**) Ascorbate peroxidase (APX), (**f**) Proline. According to the Duncan test, different letters indicate a significant difference (*p* < 0.05) among the treatments. The error bars represent the mean ± SE.

Salt and copper toxicity both induce oxidative stress in plants by overproduction of reactive oxygen species (ROS), i.e., O_2_^•−^, ^•^OH, or H_2_O_2_ [75,76]. In this study, we found that the H_2_O_2_ production increased in both the single and combined stress treatments of S (110.48%), Cu (95.9%), and SCu (125.56%), compared to CK (*p* < 0.05). However, melatonin, in single and in combination with stress treatments, reduced H_2_O_2_ production in M (19.36%), SM (40.25%), CuM (31.51%), and SCuM (34.44%), compared to CK, S, Cu, and SCu treatments, respectively (*p* < 0.05) (Figure 2a; Supplementary File S1). It has been reported that melatonin directly reacts with H_2_O_2_ and neutralizes its effects which increases the protection against ROS in plants [77,78,79].

Plant enzymatic and non-enzymatic antioxidants, including superoxide dismutase (SOD), peroxidases (POD), catalase (CAT), ascorbate peroxidase (APX), and proline, protect the plant from oxidative damage by scavenging ROS [79,80]. Pitaya seedlings under different treatments of M, S, and Cu developed sophisticated mechanisms of antioxidant activities to modulate ROS homeostasis. The SOD, POD, and CAT activities showed significant increases in the S (17.21%, 50%, and 66.53%), Cu (24.7%, 54%, and 45.35%), and SCu (16.68%, 45%, and 79%) treatments, respectively, compared to CK. The M treatment, in combination with S, Cu, and SCu, further increased SOD, POD, and CAT activities in SM (10.64%, 10.91%, and 17.04%), CuM (10.51%, 18.64%, and 16.53%), and SCuM (8.98%, 11.41%, and 9.63%), compared to S, Cu, and SCu, respectively (*p* < 0.05). The APX activity increased in S (52.74%), Cu (31.73%), and SCu (41.44%), and decreased in SM (24.76%), CuM (14.19%), and SCuM (24.67%), compared to S, Cu, and SCu, respectively (*p* < 0.05). The proline level exhibited an increase in response to M (105.93%), S (481.79%), Cu (423%), and SCu (619%), compared to CK. The melatonin application elevated the level of proline in SM (20.93%), CuM (25.18%), and SCuM (15.22%), compared to S, Cu and SCu, respectively (*p* < 0.05). There were no obvious effects of SOD, POD, CAT, and APX activities under the single M treatment. However, the proline content increased in the M treatment by 105.93%, compared to CK (Figure 2b–f; Supplementary File S1). It has been reported that SOD catalyzes the dismutation or disproportionation of superoxides (O_2_^•−^, ^•^OH) into H_2_O_2_ [81]. Further, POD, CAT, and APX degrade H_2_O_2_ to generate H_2_O [82]. Whereas, proline maintains the osmotic adjustment and also enhances antioxidant enzymatic activities [83,84]. Melatonin promotes stress tolerance in plants by enhancing plant antioxidant activities. Additional studies have shown that an exogenous application of melatonin improved antioxidant activities in white beans [85] and soya bean [86] under salt stress and in lemon balm [87] and tomato [88] under heavy metal stress.

### 2.3. Effect of Abiotic Stress Treatments on Pitaya Photosynthetic Pigments

**Figure 3:**
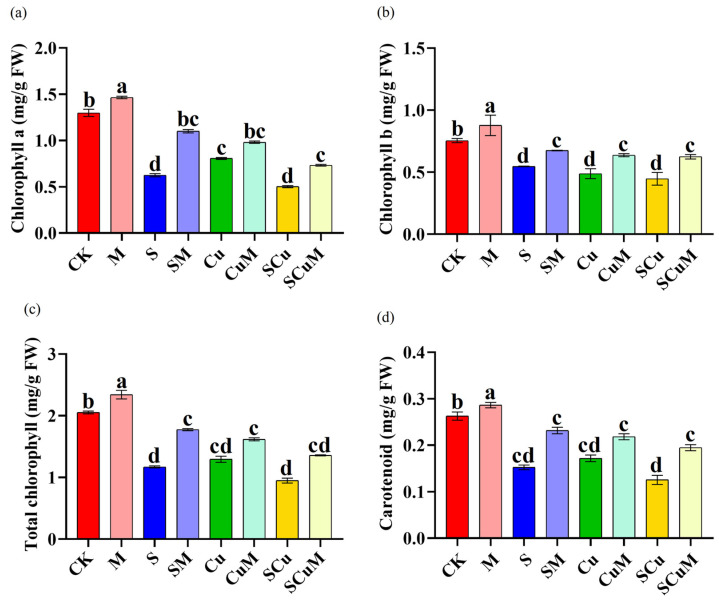
Single and combined effect of melatonin (M), salt (S), and copper (Cu) on pitaya photosynthetic pigments. (**a**) chlorophyll *a*, (**b**) chlorophyll *b*, (**c**) total chlorophyll, (**d**) carotenoid. According to the Duncan test, different letters indicate a significant difference (*p* < 0.05) among the treatments. The error bars represent the mean ± SE.

Photosynthetic pigments, chlorophyll *a*, chlorophyll *b*, and carotenoids were significantly decreased in pitaya seedlings under different stress treatments. However, melatonin effectively reversed this detrimental effect and enhanced photosynthetic pigments in the single M treatment, as well as in combination with stress treatments. The content of chlorophyll *a* decreased in S (51.85%), Cu (37.89%), and SCu (61.36%), compared to CK; and increased in M (12.83%), SM (75.97%), CuM (21.72%), and SCuM (46.1%), compared to CK, S, Cu, and SCuM, respectively (*p* < 0.05). The content of chlorophyll *b* decreased in S (27.60%), Cu (35.34%), and SCu (40.70%), compared to CK; and increased in M (16.22%), SM (23.45%), CuM (30.60%), and SCuM (39.54%), compared to CK, S, Cu, and SCuM, respectively (*p* < 0.05). Similarly, the content of total chlorophyll and carotenoid decreased in the S (42.93% and 41.97%), Cu (36.95% and 34.65%), and SCu (53.76% and 52.29%) treatments, compared to CK; and increased in M (14.07% and 8.94%), SM (53.76% and 51.86%), CuM (25.07% and 27.08%), and SCuM (43.03% and 55.36%), compared to CK, S, Cu, and SCu, respectively (*p* < 0.05) (Figure 3; Supplementary File S1). The M application, in single as well as in combined treatments, sufficiently enhanced photosynthetic pigments in pitaya. It has been reported that oxidative stress caused by salt and copper toxicity interferes with chloroplast and thylakoid membrane composition, which results in the reduction of photosynthetic pigment content [89,90]. Other studies have revealed significant decreases in chlorophyll *a*, *b*, as well as a decrease in carotenoid content in lentils treated with CuSO_4_ [91] and pigeon peas treated with NaCl [92].

### 2.4. RNA-Seq Quality Control Assessments

**Figure 4:**
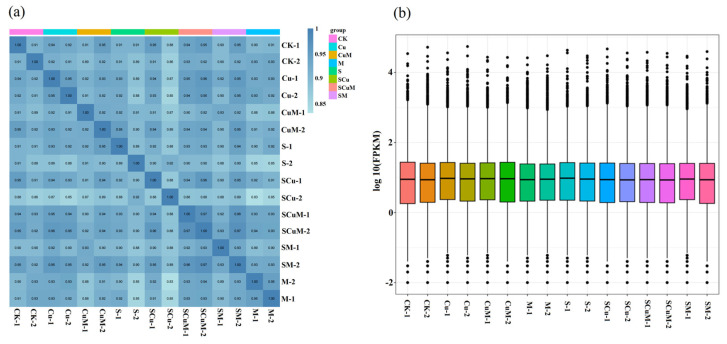
RNA−sequencing overview of pitaya seedling samples under single and combined treatments of control (CK), melatonin (M), salt (S), and copper (Cu). (**a**) Correlation heatmap shows the gene expression levels between both the replicates of treatment, including CK, M, S, Cu, SM, CuM, SCu, and SCuM. (**b**) Boxplot shows differences between the samples of both replicates using log10 transformation of FPKM values.

Plants respond simultaneously to environmental stresses and exhibit opposing strategies to mitigate any adverse effects [93]. The combination of stresses significantly affects plant growth and productivity compared to individual stresses. An increase in stress factors decreases the growth and survival of plants even if the stress levels are low [94]. To study the response of pitaya to the saline and heavy metal environment, we subjected the treated samples, including CK, M, S, Cu, SM, CuM, SCu, and SCuM, to transcriptome sequencing (DESeq2) to better understand the transcriptional dynamics under each treatment. A total of 555.56 million raw reads were obtained by an MGI high-throughput sequencer with an average of 34.72 million reads; each library had 19.63–41.68 million raw reads (Supplementary File S2). Then, we processed raw reads to filter out adapter sequences, unmeasured bases, and low-quality reads. A total of 554.89 (99.87%) million clean reads were obtained ranging from 19.61–41.63 million, with an average 36.16 million. The sequencing data generated a high-quality read with the Q20 base rate ranging from 97.57–99.075%, the Q30 base rate ranging from 92.57–96.24%, and the GC content ranging from 45.13–45.92% (Supplementary File S2). Further, the quality control (QC) clean reads were aligned with the reference genome of *S. undatus* with ~527.93 (95.14%) million total reads mapped to the genome. Approximately 470.68 (84.82%) million reads were uniquely mapped to the transcript, ranging from 16.78–31.20 with an average of 29.41 million reads per transcript (Supplementary File S2). These results indicate that the sequenced data was suitable for further analysis. In total, we identified 28,947 genes, of which 24,268 were known and 4679 were novel. We analyzed the gene expression patterns for all eight treatments, including CK, M, S, SM, Cu, CuM, and SCuM, which show a high correlation among the biological replicates (Figure 4a). A comparison of the gene expression level of each sample showed a significant difference between the different treatments (Figure 4b).

### 2.5. Differential Gene Expression Analysis Under Single and Combined Treatments

**Figure 5:**
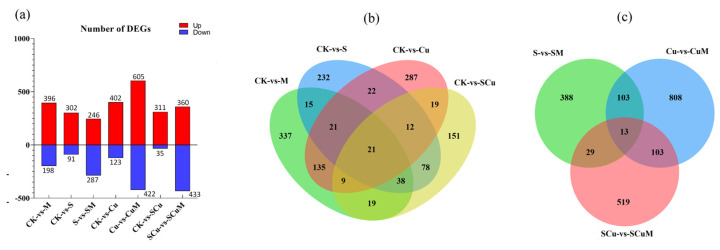
DEGs−based analysis under single and combined treatments, including control (CK), melatonin (M), salt (S), and copper (Cu). (**a**) Number of DEGs in the treatments M, S, Cu, and SCu compared to CK and SM, CuM, and SCuM compared S, Cu, and SCu, respectively. Red bars show upregulated DEGs, and blue bars shows downregulated DEGs. (**b**,**c**) Venn diagram shows the number of common and unique genes in different treatments comparison as: (**b**) CK-vs-M/CK-vs-S/CK-vs-Cu/CK-vs-SCu and (**c**) S-vs-SM/Cu-vs-CuM/SCu-vs-SCuM.

In response to stress, the plant defense system integrates signals to regulate gene expression differentially, which enhances stress tolerance against the specific stress [14]. However, melatonin also regulates gene expression and promotes stress tolerance in plants [95]. To study the unique genetic response of pitaya towards abiotic stresses and the role of melatonin in regulating stress responsive genes, we performed a pairwise comparison of gene expression levels between the treatments and control. In total, we identified 5259 (4194 known and 1065 novels; 2654 up and 1540 down) differentially expressed genes (DEGs) in all the treatments, including M, S, Cu, SCu, SM, CuM, and SCuM. We found the highest number of DEGs in CuM (1027), compared to Cu, and the lowest in SCu (346), compared to CK (Figure 5a; Supplementary File S3).

In plants, multiple genes are involved in regulating single stress [96], or a single gene regulates multiple stresses; for example, the overexpression of the *OsLEA4* gene has been shown to enhance salt, drought, and heavy metal stress tolerance in transgenic rice [97]. To identify single and multiple stress responsive genes in pitaya, we performed a comparative analysis among the DEGs of M/S/Cu/SCu to CK and SM/CuM/SCuM to S/Cu/SCu, respectively. We identified 21 common DEGs in the CK-vs-M/CK-vs-S/CK-vs-Cu/CK-vs-SCu comparison and 13 common DEGs in the S-vs-SM/Cu-vs-CuM/SCu-vs-SCuM comparison, revealing the unique or multiple roles of genes against different stresses (Figure 5b,c; Supplementary File S3). Moreover, to explore the role of melatonin in gene regulation against S, Cu, and SCu stresses, we performed a comparison between the DEGs of stress treatments, stress in combination with M, and M in single as follows: S/SM/M (64 common DEGs), Cu/CuM/M (38 common DEGs), and SCu/SCuM/M (71 common DEGs), compared to CK (Figure S2a). Furthermore, we performed a comparative expression analysis of the common DEGs to highlight the expression pattern of stress responsive DEGs and the effect of melatonin on the expression patterns under different treatments. We found significant differences in the expression level of DEGs under single and combined treatments of stress and M (Figure S2a). Considering the phenotypic results and the DEGs, analysis results predicted the significant role of melatonin in the regulation of stress response genes in pitaya, as the previous studies have reported the promising role of melatonin in the stress tolerance of diverse horticultural crops [98].

### 2.6. Gene Ontology Enrichment Analysis of DEGs Under Single and Combined Treatments

In plants, a number of genes function in several biological processes (BP) in which a gene or a gene product contributes in chemical or physical transformation, i.e., cell growth, maintenance, or signal transduction; molecular functions (MF) in which genes involved in biochemical activities, i.e., enzymes, transporters, or ligand and cellular components (CC) representing the cell, where gene product is active, i.e., the gene products in nuclear membrane or Golgi apparatus [99]. To identify gene functional annotation in pitaya under the treatments including M, S, SM, Cu, CuM, SCu, and SCuM, we performed a gene ontology (GO) enrichment analysis of the DEGs. Our analysis encompasses the top significant 20 GO terms for each treatment based on the *p*-value and Q-value. Most of the GO terms were associated with BP class, with the highest number of DEGs followed by MF and CC classes (Supplementary File S4).

**Figure 6:**
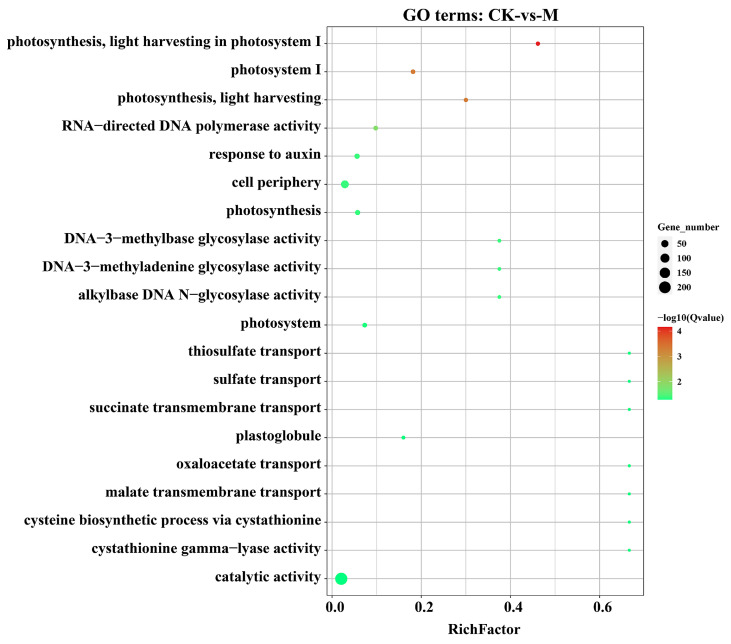
Gene ontology enrichment analysis of the DEGs under melatonin (M) treatment compared to control (CK). The size of circular dots shows the number of DEGs in each GO term. The color bar from green (min.) to red (max.) shows the enrichment of GO terms based on the Q-value of the DEGs.

Among the top 20, we found that the GO terms associated with photosynthesis (GO:0009768, GO:0009765, GO:0009522, GO:0015979), response to auxin (GO:0009733), cell periphery (GO:0071944), RNA directed DNA polymerase activity (GO:0003964), and catalytic activity (GO:0003824) are highly significant in the M treatment (Figure 6). In the S treatments, the highly significant GO terms were mostly associated with extracellular region (GO:0005576), cell wall (GO:0005618), catalytic, metabolic and enzymatic activities (GO:0003824, GO:0009698, GO:0005975, GO:0016829, GO:0004497), immune and defense responses (GO:0002376, GO:0006955, GO:0006952, GO:0006952, GO:0098542, GO:0001906, GO:0031640), and heme binding (GO:0020037). In the S-vs-SM treatment, the highly significant GO terms include catalytic activity (GO:0003824), metabolic processes (GO:0005975, GO:0005977, GO:0006112, GO:0006073, GO:0044264, GO:0044042, GO:0044262, GO:0005976, GO:0044247), enzymatic activities (GO:0016798, GO:0052793), and chloroplast and plastid stroma (GO:0009570, GO:0009532) (Figure S3a,b).

In the Cu treatment, the highly significant GO terms include enzymatic and catalytic activities (GO:0016798, GO:0003824, GO:0004553), extracellular region (GO:0005576), DNA replication and metabolic process (GO:0006260, GO:0006270, GO:0032993, GO:0140097, GO:0006259, GO:0006261, GO:0044815, GO:0006268), and photosynthesis, light harvesting in photosystem I (GO:0009768). In the Cu-vs-CuM treatment, the GO terms, including phenylpropanoid biosynthetic and metabolic process (GO:0009699, GO:0009698), cell periphery (GO:0071944), jasmonic acid metabolic process (GO:0009694), secondary metabolite biosynthetic process (GO:0044550), oxidoreductase and catalytic activities (GO:0016491, GO:0003824), molecule binding (GO:0043168, GO:0036094, GO:0019842), and flavone metabolic and biosynthesis processes (GO:0051552, GO:0051553, GO:0051554, GO:0051555), were found highly significant (Figure S3c,d). In the SCu treatment, the significant GO terms include extracellular region (GO:0005576), external encapsulating structure (GO:0030312), cell wall (GO:0005618), cell periphery (GO:0071944), phenylpropanoid biosynthesis and metabolic process (GO:0009698, GO:0009699), defense response (GO:0006952, GO:0009607), and lignin and secondary metabolites (GO:0009808, GO:0019748, GO:0044550). In the SCu-vs-SCuM treatment, the highly significant GO terms were mainly associated with the photosynthesis process (GO:0009765, GO:0009768, GO:0009522), rhythmic process (GO:0048511), cell periphery (GO:0071944), phenylpropanoid biosynthesis and metabolic process (GO:0009699, GO:0009698), lignin and pectin biosynthesis, metabolic and catabolic process (GO:0009809, GO:0009808, GO:0045490), and response to stress (GO:0006950) (Figure S3e,f).

### 2.7. KEGG Enrichment Analysis of DEGs Under Single and Combined Treatments

Multiple biological pathways are involved in managing stress responses in plants, depending on the type of stress and plant species [100]. Studies have shown that ABA-regulatory pathways improve drought tolerance in *Medicago truncatula* [101] and glyoxalase pathways contribute in detoxification under salinity and heavy metal stresses in tobacco [102,103]. To identify the significant pathways involved in pitaya abiotic stress responses, we performed a Kyoto Encyclopedia of Genes and Genomes (KEGG) annotation and enrichment analysis of the DEGs identified in each treatment including M, S, SM, Cu, CuM, SCu, and SCuM compared to CK. We encompassed the most significant top 20 highly enriched pathways in each treatment based on *p*-value and Q-value (Supplementary File S5).

**Figure 7:**
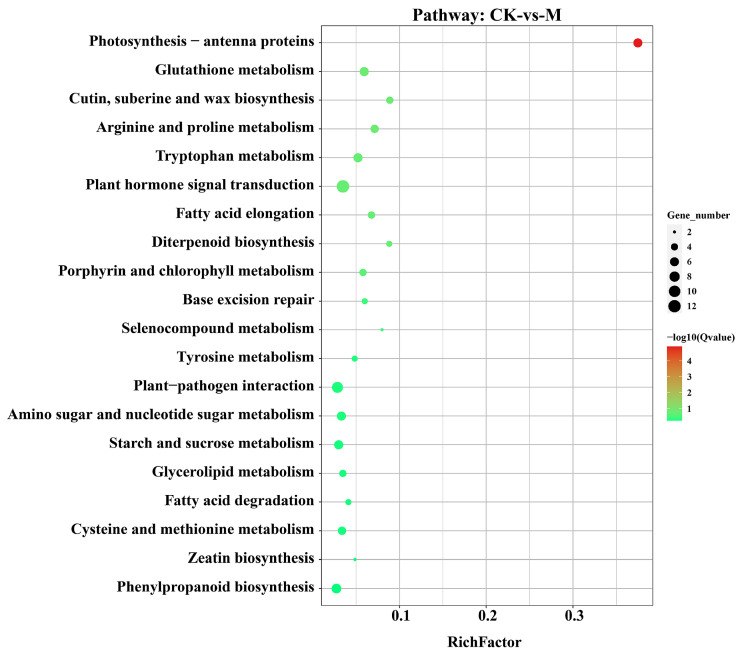
The Kyoto Encyclopedia of Genes and Genomes annotation and enrichment (KEGG) analysis of the DEGs identified in each melatonin (M) treatment compared to control (CK). The size of circular dots shows the number of DEGs in each pathway. The color bar from green (min.) to red (max.) shows the enrichment of pathways based on the Q-value of the DEGs.

Among the top 20, we found that the photosynthesis-antenna protein, glutathione metabolism, cutin, suberine and wax biosynthesis, arginine and proline metabolism, tryptophane metabolism, and plant hormone signal transduction pathways were highly significant in the M treatment (Figure 7). In the S treatment phenylpropanoid biosynthesis, cutin, suberine and wax biosynthesis, carbon fixation in photosynthetic organisms, flavonoid biosynthesis, and starch and sucrose metabolism pathways were highly enriched. Whereas the melatonin application in S-vs-SM treatment enriched the pathways including carbon fixation in photosynthetic organisms, starch and sucrose metabolism, MAPK signaling pathway–plant, glycolysis/gluconeogenesis and flavonoid biosynthesis pathway (Figure S4A,B). In the Cu treatment, the enriched pathways included DNA replication, photosynthesis-antenna, phenylpropanoid biosynthesis, mismatch repair, base excision repair, nucleotide excision repair, and glutathione metabolism pathways. Whereas, in the Cu-vs-CuM treatment, melatonin enhanced the enrichment of phenylpropanoid biosynthesis, MAPK signaling pathway-plant, cysteine and methionine metabolism, alpha-Linolenic acid metabolism and cutin, and suberine and wax biosynthesis pathways (Figure S4C,D). Similarly, in the SCu treatment, the highly enriched pathways include cutin, suberine and wax biosynthesis, phenylpropanoid biosynthesis, glycerolipid metabolism, and plant hormone signal transduction pathways. Whereas the melatonin combination in the SCu-vs-SCuM treatment enhanced the photosynthesis-antenna proteins, cysteine and methionine metabolism pentose, and glucuronate interconversions and MAPK signaling pathway (Figure S4E,F).

Additionally, we observed that the differential gene expression in a respective pathway depends on the stress factor. In pitaya, a total of 257 genes are involved in phenylpropanoid biosynthesis pathways, but only 7, 20, 12, 25, 13, and 10 genes were differentially expressed under the M, S, SM/Cu, CuM, SCu, and SCuM treatments, respectively. Similarly, differences in the DEGs were also observed in other significant pathways under different treatments. We also observed that genes have multiple functions and play a role in the regulation of different pathways; for example, the HU06G00874 gene is expressed differentially under the S treatment and plays a role in the flavonoid biosynthesis, phenylpropanoid biosynthesis, stilbenoid, diarylheptanoid, and gingerol biosynthesis pathways (Supplementary File S5). These results show that several pathways were involved in regulating stress tolerance in pitaya under the single and combined stress factors of S and Cu, as well as in combination with M. The melatonin application enhanced the regulation of some stress responsive pathways, including MAPK signaling pathways, tryptophan metabolism, phenylpropanoid biosynthesis, cutin, suberine, and wax biosynthesis under different treatments. Some pathways were found common in all treatments, including phenylpropanoid biosynthesis, amino sugar and nucleotide sugar metabolism, and tryptophan metabolism pathways.

### 2.8. Transcriptional Factors Involved in Pitaya Abiotic Stress Responses

The genome assigns approximately 7% of the coding region to transcriptional factors (TFs), which are the key regulators of gene expression to develop stress resistance in plants [104]. In pitaya, we studied the DEGs involved in the transcription regulation process using the plant transcription factor database—PlantTFDB 4.0 [105]. In all the treatments, we identified a total of 1024 transcriptional factor genes distributed in 38 families. The majority of TF genes were found in *S1Fa-like*, *MYB* and *MYB*_related, *RAV*, *GATA*, *AP2/ERF*, *CAMTA*, *bHLH*, *NAC*, *GRAS*, and *WRKY* gene families (Figure S5). Among the 1024, only 44 TF genes with 97 transcripts were found to be involved in pitaya development and stress responsive pathways. These include members of *S1Fa-like* (HU02G03030, HU06G01950, HU09G01085, HU09G01540 HU02G03030, HU02G00526, HU02G02103, HU02G01338), members of *AFR* (HU05G02286, HU03G00685, HU03G02449, HU11G00661, HU04G01928, HU03G02995, HU03G00626), members of *ERF* (HU06G02316, HU04G01825), members of *WRKY* (HU04G01010, HU09G01037, HU02G01423), members of *bHLH* (HU04G00514, HU04G00121), members of *GATA* (HU02G00526, HU02G02103), and a member of *ARR-B/CO*-like (HU04G01777) involved in plant–pathogen interaction, MAPK signaling pathway—plant, plant hormone signal transduction, and circadian rhythms—and plant pathways under different treatments (Supplementary File S6). Previous studies have reported that the overexpression of *S1Fa-like* TF family members (*PtS1Fa1* and *PtS1Fa2*) in *Populus trichocarpa* [106], a member of *bHLH* (*AtbHLH39* and *AtbHLH104*) in Arabidopsis [107], a member of *WRKY* (*VvWRKY2*) in potato [108], and a member of *AP2/ERF* (*SlERF1*) in tomato [109] enhanced drought, salt, cold, and heavy metal tolerance. This evidence reveals the role of *S1Fa-like*, *WRKY*, *bHLH*, and *ERF* TFs in regulating S and Cu stress responses in pitaya.

### 2.9. MAPK Signaling Pathway Involvement in Pitaya Abiotic Stress Tolerance

The mitogen-activated protein kinases (MAPK) pathway is a key element in plant defense responses under biotic and abiotic stresses. In plants, it is activated by external stimuli via three layers of protein kinases (*MAPKKKs*→*MAPKKs*→*MAPKs*) which are enrolled in signaling, consisting of interacting proteins and activation by phosphorylation. The phosphorylated MAPKs translocate to the nucleus from cytoplasm which regulate gene expression [110]. In pitaya, the MAPK signaling pathway is found to be highly significant, and several MAPK-related genes were differentially expressed under various treatments (Supplementary File S7). Under S, Cu, and SCu treatments *PR1* (HU01G00526), *ERF1* (HU04G01825, HU06G02316), *ChiB* (HU11G01456, HU06G01980), *RbohD* (HU08G01494, HU03G02488, HU11G00409), and *ER/ERLs* (HU02G03030, HU09G01540) genes were upregulated compared to CK. It has been reported that the *PR1* gene encodes the pathogenesis-related protein 1, which enhances resistance to both biotic and abiotic stresses [111]. The overexpression of rice *OsPR1a* and pepper *CABPR1* genes in Arabidopsis enhanced salt and heavy metal tolerance in transgenic Arabidopsis [112,113]. The *ERF1* gene expression regulates *ChinB* which encodes chitinase. Chitinases are involved in various abiotic stress responses, i.e., osmotic pressure, cold, salt, and heavy metal stress. The over expression of *ERF1* (*HbERF-IXc5*) enhanced salt and cold stress tolerance in *Hevea brasiliensis* [114]. *RbohD* regulates ROS signaling networks and plays an essential role in various stress responses. In Arabidopsis, the *AtRBOHD/F* gene regulates ion homeostasis in response to salt stress [115]. *ER/ERLs* genes function in plant development, such as stomatal function, pathogen attack, and senescence, under various stress conditions [116]. Under the SM treatment, when compared to S, melatonin enhanced the expression of *PR1* (HU10G00914) and *PP2C* (HU02G01597, HU05G01575). The *PP2C* genes are involved in various signaling pathways and enhance stress resistance in plants. Overexpression of the rice *PP2C* (OsPP108) gene in Arabidopsis enhanced salt tolerance and drought tolerance in transgenic Arabidopsis. Whereas the *MKK9* genes (HU06G02342, HU06G02360) were downregulated in the SM treatment. *MKK9* has been reported as a negative regulator in Arabidopsis under salt stress [117,118]. Under the CuM treatment, when compared to Cu, melatonin enhanced the expression of the *MPK1/2* (HU05G02055) and *NDPK2* (HU02G00959) gene associated with H_2_O_2_ signaling in pitaya. In *Brassica napus*, the *BnMAPK1* gene was overexpressed, which enhanced antioxidant activities and drought tolerance [119]. The *NDPK2* gene in Arabidopsis enhanced tolerance to oxidative stress by activating H_2_O_2_-dependent MAPK signaling [120]. Under the SCu treatment, the *CaM* (HU06G02191, HU09G01996) genes were upregulated and involved in calcium based signaling in response to the S and Cu combined treatment. It has been reported that in Arabidopsis the *CaM* genes (*AtCaM4* and *AtCaM2*) regulate salt and heavy metal tolerance [121,122]. Under the SCuM treatment, melatonin enhanced *CAT1* (HU05G00832) gene expression compared to SCu. The *CAT1* gene encodes catalases which act as an antioxidant against H_2_O_2_ and enhance the oxidative stress tolerance in plants [123].

### 2.10. Plant Hormonal Signaling Pathway in Pitaya Abiotic Stress Tolerance

Phytohormones (PHs), such as auxin, cytokinins, ethylene, brassinosteroids, gibberellins, jasmonates, and salicylic acid, are the important regulators of plant growth, development, and environmental responses. Their signaling depends on their distribution and activity of regulators. Their associated gene expression depends on genetically encoded phytohormone signaling manipulators (GEPHMans) [124]. PHs act as chemical messengers and regulate multiple stress responses in plants [125]. In pitaya, melatonin enhanced the *AUX/IAA* (HU05G02286, HU03G00685) and *SAUR* (HU03G00208, HU03G00207, HU01G00211) gene expression in the M treatment. *Aux/IAA* proteins regulate auxin-induced gene expression and work as antennae to connect auxin and light signals to endogenous developmental responses [126]. *SAUR* proteins are involved in auxin-regulated cell expansion. Overexpression of *SAUR36* and *SAUR41* increase cell expansion via hypocotyl elongation [127,128]. Under the S treatments, the *GH3* (HU06G00395) gene was upregulated associated with auxin. *GH3* functions in auxin homeostasis by conjugating excess indole-3-acetic acid (IAA) to amino acid. The knocking out of the *GH3* gene (*gh3oct*) in Arabidopsis increased the IAA-dependent tolerance to salinity [129]. Under the SM treatment, when compared to S, *PP2C* (HU02G01597, HU05G01575) was upregulated. Under the Cu treatment, the *CYCD3* (HU01G00165) gene is upregulated, which functions in brassinosteroid production and signaling and enhances tolerance to oxidative stress [130]. Under the CuM treatment, when compared to Cu, the *ARF* (HU03G02449) gene is upregulated, which functions in auxin signaling. The expression of *ARF* regulates various developmental and abiotic stress responses in plants, including heavy metals [131]. Under the SCu treatment, *JAZ* (HU02G00566) is upregulated encoding the jasmonate *ZIM* domain-containing protein. It has been reported that jasmonate regulates various stress responses in tomatoes [132]. Under the SCuM treatment, the *LAX* (HU07G00309) gene in auxin signaling and the *BSK* (HU02G01338) gene in brassinosteroid signaling were upregulated. Whereas the *GH3* (HU01G01860, HU06G00394) gene was downregulated (Supplementary File S7).

### 2.11. Phenylpropanoid Biosynthesis Pathway in Pitaya Abiotic Stress Tolerance

Phenylpropanoids are the plants bioactive secondary metabolites, categorized into five different groups, including flavonoids, lignin, phenolic acids, stilbenes, and coumarins [133]. They play a diverse role in plant growth, development, and adaptation to various abiotic stresses, i.e., salinity, heavy metals, drought, UV radiations, heat, and cold [134]. According to the KEGG analysis in pitaya, the phenylpropanoid biosynthesis pathway is found highly significant in all the treatments, including M, S, SM, Cu, CuM, and SCuM (Supplementary File S7). Under the M treatment, *PAL* (HU09G00737) was upregulated encoding phenylalanine ammonia-lyase, which catalyzes the first step in the phenylpropanoid pathway, promoting the biosynthesis of various secondary metabolites [135]. *HTC* (HU10G00264) is upregulated under the M treatment, which encodes shikimate O-hydroxycinnamoyltransferase enrolled in plant lignin and secondary cell wall biosynthesis. The silencing of the *HTC* gene in Arabidopsis resulted in a strong reduction in plant growth [136]. Under the S treatment, the *HCT* (HU10G00264), *4CL* (HU09G00026), *CCR* (HU01G00588), *COMT1* (HU06G01469, HU02G01791), and *POD* (HU06G01090, HU06G01090, HU02G02575) genes were upregulated. A previous study has reported that the *4CL* gene encodes 4-coumarate-CoA ligase and the *CCR* genes encode cinnamoyl-CoA reductase, which is a key enzyme in lignin and flavonoid biosynthesis [137]. Lignin plays a crucial role in plant development, cell osmotic balance, and protect cell membrane integrity under high salinity [138]. Whereas, flavonoids are the major non-enzymatic antioxidants and protect the plants from oxidative stress caused by salinity and other stresses [139]. The *COMT1* gene encodes caffeic acid 3-O-methyltransferase and functions in the biosynthesis of endogenous melatonin [140]. POD acts as an antioxidant and degrades H_2_O_2_ to generate H_2_O, as discussed in Section 2.2 [79,141]. Under the SM treatment, the *COMT1* genes (HU06G01469, HU02G01791, HU09G00002, HU09G00064) were downregulated, when compared to S, suggesting that the exogenous application of melatonin minimizes its endogenous biosynthesis in pitaya and, thus, the *COMT1* gene expression is downregulated in the SM treatment. Under the Cu treatment, *HTC* (HU10G00264), *COMT* (HU09G00004), *POD* (HU09G00247, HU03G01694, HU08G01569), and *BGLU* (HU05G00795) were highly upregulated; whereas, some *POD* (HU06G01090, HU06G00913) genes were downregulated. The *BGLU* gene encodes beta-glucosidase which plays a crucial role in cell wall lignification in response to copper stress. It has been reported that beta-glucosidase shows high resistance to soil contaminated by copper [142]. Under the CuM treatment, when compared to Cu, the *PAL* (HU06G02082), *4CL* (HU09G00026), (HU03G00517), and *POD* (HU05G01965, HU09G00243, HU06G00913, HU06G01090, HU02G02575) genes were upregulated. Under the SCu treatment, the *4CL* (HU06G01469, HU02G01791), POD (HU03G01694, HU10G00510, HU09G00059), *COMT1* (HU06G01469, HU02G01791), CYP84A (HU05G02075), and CCoAOMT (HU06G00874, HU01G00147) genes were upregulated. Whereas, under the SCuM treatment, when compared to SCu, only *TOGT1* (HU10G00514) was upregulated. *TOGT1* encodes scopoletin glucosyltransferase, which plays a key role in hormonal homeostasis, detoxification, and the biosynthesis of secondary compounds [143]. The *CCoAOMT* gene encodes caffeoyl-CoA O-methyltransferase, which plays a significant role in lignin synthesis. It has been reported that lignin synthesized by the *CCoAOMT* gene showed high tolerance to Cu toxicity in rice [144].

### 2.12. Single and Combined Stresses and Melatonin Application Regulated Photosynthesis Related Genes

Photosynthesis is the main energy production process and driving force for plant growth and biomass production. Chloroplast plays an important role in capturing light energy to assimilate carbon dioxide (CO_2_) and the water required for the biosynthesis of the developmental organic compounds [145,146]. Under the M treatment, the *PsaO* (HU07G02067), *LHCA* (HU02G02938, HU01G01505, HU01G02401), and *LHCB3* (HU0G00080, HU01G01905, HU11G01748) genes were upregulated. The *LHCA* and *LHCB* genes encode light-harvesting complex proteins which bind to chlorophyll and carotenoids for energy production via photosystem I (PSI) and II (PSII), respectively [147]. Whereas, the *PsaO* gene regulates the excitation pressure between PSI and PSII [148]. Under the S treatment, the *GAPDH* (HU04G01247), *PPC* (HU01G00988, HU01G00987, HU01G00988, HU01G00987) and *PGK* (HU06G01772) genes were downregulated; while the melatonin application enhanced their expression in the SM treatment when compared to S. The *GAPDH*, *PPC*, and *PGK* genes function in carbon fixation and metabolism in the photosynthesis process [149,150]. Under the Cu treatment, the *PPC* (HU01G00989) gene was downregulated; and under the CuM treatment, when compared to Cu, *MDH* (HU06G00916) and *maeB* (HU03G01724) were upregulated. *MDH* catalyzes the conversion of oxaloacetate to malate, and *maeB* catalyzes the decarboxylation of malate to form pyruvate and releases CO_2_, which functions in the chloroplast Calvin cycle [151]. Under the SCu treatment, *FBP* (HU05G01086) was upregulated, which encodes fructose 1,6-bisphosphatase (*FBPase*). *FBPase* plays a key role in carbon metabolism and assimilation [152]. Under the SCuM treatment, the *LHCA* and *LHCB* genes were highly upregulated when compared to SCu. These results show that melatonin plays a key role in regulating the pitaya photosynthesis process and promotes pitaya growth under stress conditions (Supplementary File S7).

### 2.13. WGCNA Construction and Identification of Key Modules

**Figure 8:**
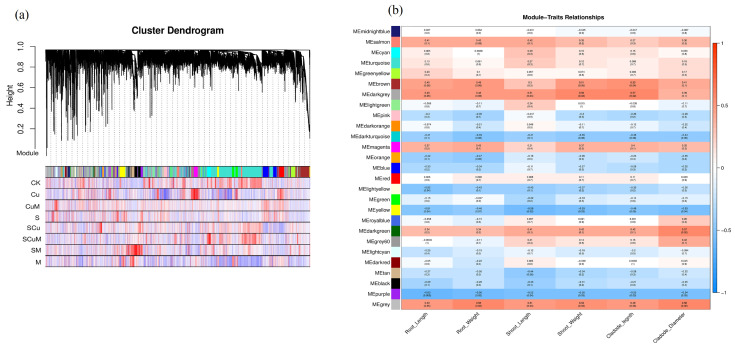
Weighted gene co-expression network analysis. (**a**) Clustering dendrogram, module construction, and correlation between modules and treatments. (**b**) Heatmap for the relationships of modules and plant traits. Each cell comprises corresponding correlations and *p*-value. Red color cells show positive correlations, and blue color shows negative correlations.

A weighted gene correlation network analysis (WGCNA) is one of the key methods to identify the potential network, significant gene clusters, intramodular hub genes, and their correlation with plant traits and treatments [153]. In this study, we performed a WGCNA analysis to identify potential networks and significant intramodular hub genes associated with measured phenotypes in pitaya under single and combined treatments of melatonin, salt, and copper, including M, S, SM, Cu, CuM, SCu, and SCuM. Low expression genes (FPKM < 1) were discarded to avoid misleading results. The threshold power was defined as 15 and the scale-free topology index was customized at 0.8 (Figure S6). Out of 28,947 DEGs, 14,473 genes were clustered and divided into 27 stable expression modules (Figure 8a). To measure the relationship between module and treatments, the criteria was set as a Pearson correlation coefficient (r = 0.5) and significance of *p*-value (*p* ≤ 0.05). The results revealed a significant correlation of brown, dark grey, dark green, and grey modules with the plant traits (RL, RW, SL, SW, CL, and CD). The genes in the brown module were highly correlated with SW and CD, dark grey with SL, SW and CL, dark green with CD, and grey with RW, SL, SW, and CD (Figure 8b).

### 2.14. GO and KEGG Enrichment Analysis of Genes in Key Modules

**Figure 9:**
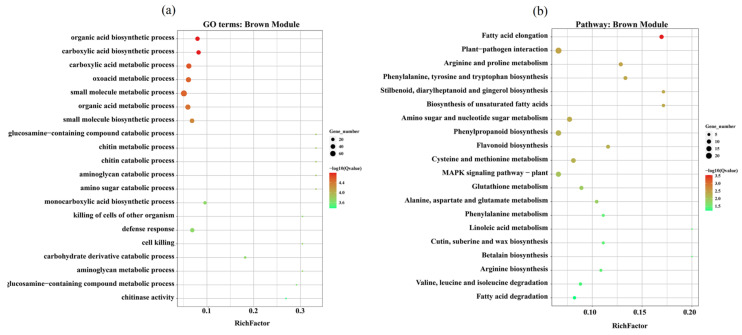
Gene ontology and the Kyoto Encyclopedia of Genes and Genomes annotation and enrichment analysis of the DEGs identified in the brown module. (**a**) Top 20 GO enrichments in the brown module. (**b**) Top 20 KEGG enrichments in the brown module. The color bar from green (min.) to red (max.) shows the enrichment of pathways based on the Q-value of the DEGs.

The GO analysis of the key modules revealed that the brown module showed significant enrichment of BP, including carboxylic acid biosynthetic process (GO:0046394), chitin metabolic and catabolic process (GO:0006030, GO:0006032), and defense response (GO:0004096). Dark green was enriched with BP, CC, and MF, including organonitrogen compound metabolic process (GO:1901564), proteasome complex (GO:0000502), endopeptidase complex (GO:1905369), scavenger receptor activity (GO:0005044) and NADPH-hemoprotein reductase activity (GO:0003958). Dark grey was enriched with BP, CC, and MF, including carbohydrate transport (GO:0008643), homocysteine metabolic process (GO:0050667), mitochondrial tricarboxylic acid cycle enzyme complex (GO:0030062), and catalase activity (GO:0004096). The grey module was enriched with CC and BP, including mitochondrial respiratory chain complex I (GO:0005747), oxidoreductase complex (GO:1990204), and proteasomal ubiquitin-independent protein catabolic process (GO:0010499) (Figure 9a and Figure S7a–c, Supplementary File S7). Whereas, the KEGG analysis showed the enrichment of fatty acid elongation, MAPK signaling-plant, phenylpropanoid biosynthesis, and plant-pathogen interaction pathways in the brown module. In the dark green module, proteasome, plant hormone signal transduction, flavonoid biosynthesis, MAPK signaling-plant and phenylpropanoid biosynthesis pathways were significantly enriched. In the dark grey module, cysteine and methionine metabolism, pyruvate metabolism, valine, leucine and isoleucine degradation, and arginine and proline metabolism pathways were significantly enriched. In the grey module, the enriched pathways include oxidative phosphorylation, purine metabolism, steroid biosynthesis, and nucleotide excision repair pathway (Figure 9b and Figure S7d,e, Supplementary File S7).

### 2.15. Network Construction and Identification of Hub Genes

**Figure 10:**
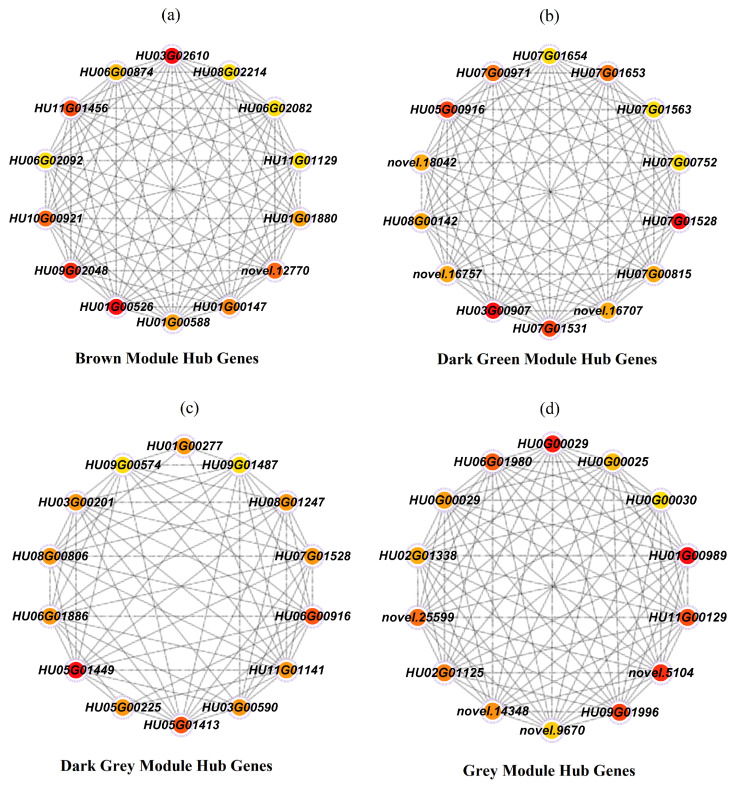
Co-expression network of hub genes. (**a**) Hub genes in the brown module. (**b**) Hub genes in the dark green module. (**c**) Hub genes in the dark grey module. (**d**) Hub genes in the grey module. Red, orange, and yellow color shows highest, moderate, and low interaction of genes. Top 14 nodes were predicted using the cytoHubba program in Cytoscape.

Gene production is implicated in a wide range of interactions and mechanisms of cellular processes. Thus, the gene products, protein–protein interactions (PPIs), and regulatory networks function in several cellular responses, such as signaling transductions, homeostasis control, and abiotic stress responses [154]. To identify the stress responsive genes in pitaya, we performed a network analysis for the brown, dark green, dark grey, and grey modules and the top 14 genes were selected as the hub genes of the network in each module (Figure 10a–d). Among the hub genes, seven genes in the brown module were identified, including *ChiB*, *PR1*, *CCoAOMT*, *PAL*, *GH3*, *CCR*, and *PTI*. *ChiB* (HU11G01456) encodes chitinase; *PR1* (HU01G00526) encodes pathogenesis-related protein 1; *CCoAOMT* (HU06G00874, HU01G00147) encodes caffeoyl-CoA O-methyltransferase and synthesize lignin; *PAL* (HU06G02082) encodes phenylalanine ammonia-lyase and catalyzing first step of phenylpropanoid biosynthesis; *GH3* (HU06G00395) functions in auxin homeostasis by conjugating excess indole-3-acetic acid (IAA) to amino acid; *CCR* (HU01G00588) encode cinnamoyl-CoA reductase which is a key enzyme in lignin and flavonoid biosynthesis; and *PTI* (HU08G02214) encodes pathogenesis-related transcriptional activators and can contribute in both biotic and abiotic stress responses [155]. Three genes were identified in the dark green module, including *EIN3*, *trpB*, and *ABF*. *EIN3* (HU03G00907) encodes ethylene related proteins and functions in salt stress tolerance [156]; *trpB* (HU08G00142) encodes tryptophan synthase enzyme; and *ABF* (HU07G00971) functions in the regulation of osmotic stress in plants [157]; Four genes were identified in the dark grey module, including *MDH*, *ACO*, *mipp1*, and *BCE2*. *MDH* (HU06G00916) encodes malate dehydrogenase; *ACO* (HU06G01886) encodes 1-aminocyclopropane-1-carboxylate oxidase; *mipp1* (HU05G01413) encodes inositol polyphosphate phosphatase; and *BCE2* (HU09G00574) encodes lipoamide acyltransferase. Three genes were identified in the grey module, including *PPC1*, *ChiB*, and *CALM*. *PPC1* (HU01G00989) encodes phosphoenolpyruvate carboxylase; *ChiB* (HU06G01980) encodes chitinase; and *CALM* (HU09G01996) encodes the CALM proteins. All 14 genes function as calcium sensors in plant cells [158] and were found to be involved in various stress responsive and growth regulating pathways. The genes in the brown module were found to be more specific to stress responsive pathways, including MAPK signaling pathway—plant, amino sugar and nucleotide sugar metabolism, plant hormone signal transduction, plant–pathogen interaction, flavonoid biosynthesis, phenylpropanoid biosynthesis and stilbenoid, and diarylheptanoid and gingerol biosynthesis.

### 2.16. Comparative Data Set Analysis to Identify the Potential Candidate GENES from RNA-Seq Data

To identify potential candidate genes in response to single and combined treatments of M, S, and Cu in pitaya, we compared the stress responsive DEGs identified by DESeq2 analysis under all treatments and genes from the WGCNA trait modules analysis. By comparing both analyses, we found 12 DEGs common and highly correlated to stress treatments and plant traits, distributed in the brown, dark green, dark grey, and grey modules. A comparative study revealed that the *GH3* (HU06G00395), *JAZ* (HU02G00566), *PAL* (HU09G00737, HU06G02082), *CCR* (HU01G00588), *POD* (HU10G00510), *CCoAOMT* (HU06G00874, HU01G00147), *PPC* (HU01G00989), *PR1* (HU01G00526), *ChiB* (HU11G01456), and *MDH* (HU06G00916) genes were differentially expressed under various treatments and highly correlated with the pitaya traits.

Furthermore, our findings predicted that *GH3* (HU06G00395), *POD* (HU10G00510), *CCoAOMT* (HU06G00874), and *ChiB* (HU11G01456) were multiple stress responsive genes and highly expressed under the S and SCu treatments when compared to CK. *CCR* (HU01G00588) and *PPC* (HU01G00989) are single stress responsive and expressed under S and Cu, respectively, when compared to CK. The M application highly expressed *PAL* (HU06G02082), *CCoAOMT*, (HU01G00147), and *MDH* (HU06G00916) gene expression under the CuM treatment when compared to Cu. Whereas, the M application downregulated the expression of the *JAZ* (HU02G00566), *CCoAOMT* (HU06G00874) and *PR1* (HU01G00526) genes under SCuM treatment when compared to SCu (Supplementary File S10). These results predict the potential role of these candidate genes in pitaya self- and melatonin-mediated stress tolerance via regulating multiple stress responsive pathways.

### 2.17. Verification of RNA-Seq Data by RT-qPCR

**Figure 11:**
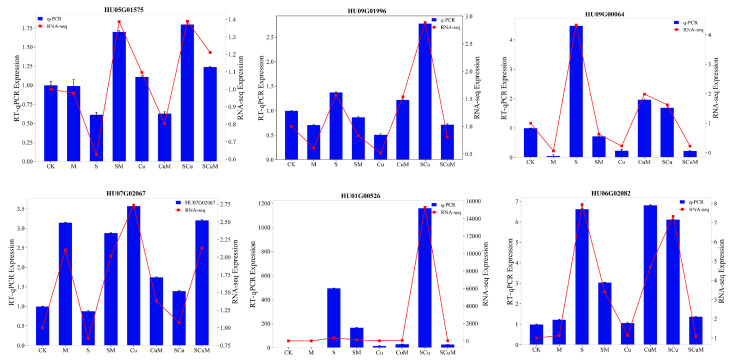
RT−qPCR analysis of candidate genes and comparison with RNA-seq. Variables at left side indicate expression of RT-qPCR and variables at right side indicate expression of RNA-seq. Blue bars show expression results of RT-qPCR and red lines with dots show expression of RNA-seq.

To verify the authenticity and reproducibility of the RNA-seq data under the CK, M, S, SM, Cu, CuM, SCu, and SCuM treatments, six candidate genes (HU07G02067, HU09G01996, HU09G00064, HU05G01575, HU01G00526, HU06G02082) were selected from both the WGCNA and DESeq2 analysis for the RT-qPCR analysis. The gene expression results of the RT-qPCR were then compared with the RNA-seq data, revealing the significant positive correlation between RNA-seq and RT-qPCR (Figure 11).

## 3. Materials and Methods

### 3.1. Plant Growth, Treatments and Phenotyping

*S. undatus* fruits were sourced from Hainan-Shengda modern agricultural development company, Qionghai, Hainan, China. The seeds were extracted and sown in controlled conditions at the Nanfan Research Institute of Hainan University, China. Initially, dragon fruit seeds were placed on petri plates containing moist filter paper for 3 days in the dark and the next 11 days in day/night conditions. The seedlings were then transferred to hydroponic pots enriched with 100% Hoagland nutrient solution [71] on the 15th day for the next 37 days. The pots were then placed in a growth chamber maintained at 16 h/8 h day/night period with 70% relative humidity for 52 days. The Hoagland nutrient solution was renewed every 2 weeks. The seedlings with uniform growth were selected for the experiment. The experiment was designed with eight different treatments as follows: control (CK), melatonin (M), salt (S), copper (Cu), S + M (SM), Cu + M (CuM), S + Cu (SCu), and S + Cu + M (SCuM) with two replicates. To obtain the optimum concentration of S, Cu, and M, a pre-experiment was conducted with different concentrations of S (NaCl: 100 mM, 150 mM, and 200 mM), Cu (CuSO_4_: 150 µM, 200 µM, and 250 µM), and M (C_13_H_16_N_2_O_2_: 50 µM, 100 µM, and 150 µM). The optimum concentration of CuSO_4_ (200 µM), NaCl (150 mM), and M (100 µM) was recorded based on the seedling phenotypical appearance (Figure S1). The seedlings were then exposed to the optimum concentration of M, S, and Cu individually and in combination for 7 days. Plant roots, stems, and cladodes were collected for morphological parameter determination, including root length (RL—cm), root weight (RW—mg), shoot length (SL—cm), shoot weight (SW—mg), cladode length (CL—cm), and cladode diameter (CD—mm), as well as antioxidant assays, chlorophyll content determination, metal ion detection, transcriptomic analysis, and real-time quantitative PCR expression analysis.

### 3.2. Sample Preparation for Copper Quantification

To quantify copper concentration accurately, 0.5 g of each pitaya plant sample with two biological replicates, treated with CuSO_4_ in single and in combination with NaCl and M were taken in a Teflon vessel. For the digestion of each sample, 5 mL nitric acid was added to each vessel containing samples and then the vessels were subjected to three successive heating programs of 120 °C, 160 °C, and 190 °C for 3, 5, and 20 min, respectively. Acid extractor was added to each vessel and placed to dry completely. Quantification of Cu in each sample was carried out by using Thermo Fisher Scientific iCAP TQ ICP-MS/MS (Thermo Fisher, Waltham, MA, USA) equipped with standard nickel cones, connected with autosampler (ASX-560, Teledyne CETAC, Omaha, NE, USA) (Zhejiang, China) [159].

### 3.3. Analysis of Oxidative Damage, Antioxidants, and Antioxidant Enzymes

The level of hydrogen peroxide (H_2_O_2_), proline, and antioxidant enzymes, including superoxide dismutase (SOD), peroxidase (POD), catalase (CAT), and ascorbic acid peroxidase (APX), were determined by utilizing the specified kits, obtained from Nanjing Jiancheng Bioengineering Institute in Nanjing, China. According to the kit’s instruction, crushed frozen cladode samples (1 g) of the two biological replicates for each treatment, as well as the control, were homogenized individually in 9 mL of PBS buffer (pH 7.4) [160]. The samples were then centrifuged at 10,000 rpm (4◦C) for 15 min and the supernatants were collected for further analysis.

The H_2_O_2_ content was determined using a titanium sulfate method [161]. The total protein content was measured utilizing the assay kit (A045-2) with absorbance readings at 959 nm. The SOD activity was determined utilizing an SOD assay kit (A001-1), and the absorbance was measured at 550 nm. POD activity determination was based on guaiacol oxidation at 479 nm, utilizing a POD assay kit (A084-3-1). The CAT activity was measured using a CAT assay kit (A007-1). Similarly, the APX activity, monitoring the oxidation of ascorbate at 290 nm, was accessed with an APX assay kit (A123-1-1) [162,163]. The level of proline was measured utilizing a proline determination assay kit (A107-1-1). The proline concentration was measured at 520 nm [164,165].

### 3.4. Determination of Photosynthetic Pigments

Chlorophyll pigments, including chlorophyll *a*, chlorophyll *b*, total chlorophyll and carotenoids concentrations, were determined. Approximately 100 mg of fresh cladode samples of the two biological replicates for each treatment, as well as the control, were homogenized with acetone (80%) and then centrifuged at 7000 rpm for 10 min. The supernatant was used for the absorbance at 663 nm for chlorophyll *a*, 646 nm for chlorophyll *b*, and 470 nm for carotenoids using an ELIDA plate reader [166,167]. Chlorophyll and carotenoids concentration was calculated as followed by [168].

### 3.5. RNA Extraction, Library Preparation, and Illumina Sequencing

Pitaya cladodes and leaves of all eight treatments with two biological replicates were used for RNA extraction. Total RNA was extracted from 0.5 g of each sample, utilizing an RNA extraction kit (VAZYME, Nanjing, China). Then the RNA of each sample was treated with DNase I enzyme to remove genomic DNA contamination. The quality and integrity of RNA was accessed through 1% agarose gel electrophoresis and a microplate spectrophotometer (BioTek Company, Winooski, VT, USA). The purified RNA was then used for the construction of cDNA libraries for each sample using an NEBNext Ultra RNA Library Prep Kit. The quality of the cDNA libraries was measured by Agilent 2100 bioanalyzer (Agilent technologies, Santa Clara, CA, USA) and sequencing was performed on the Illumina Novaseq 6000 platform by Gene Denovo Biotechnology Co., Ltd., in Guangzhou, China.

### 3.6. RNA-Seq Data Processing, Transcriptome Assembly and Gene Functional Annotation

To determine the reliability and quality control of sequenced data, the raw reads were processed using FASTQ (fq) (version 0.19.7), and Novogene personalized transcriptome pipeline. The quality-controlled clean reads were obtained using Adopt SOAPnuke (v2.1.0) [169]. The clean reads were then compared to the reference genome of *Selenicereus undatus* L. (available at: http://www.pitayagenomic.com/, accessed on 12 May 2024) using HISAT2 (2.0.5) software [170] to obtain the information of read positions on the reference genome. The obtained alignments were then processed to obtain read counts for the genes in the dragon fruit genome database [171]. The raw reads were normalized and were used for the differential expression analysis using DESeq2 (v1.20.0). The FPKM (fragments per kilobase of transcript per million mapped reads) method was used to quantify the gene expression level [172]. To perform the transcript assembly, StringTie software (version 2.2.1) was used [173]. Gene ontology (GO) and Kyoto Encyclopedia of Gene and Genomes (KEGG) pathway enrichment analyses on the differentially expressed gene sets were performed by the clusterProfiler R (4.0) package [174]. The significantly enriched GO terms were identified with Q-value ≤ 0.05. Gene enrichment analysis (GSEA) was performed using GO, KEGG, and other datasets for the differentially upregulated and downregulated genes of pitaya.

### 3.7. Genes Co-Expression Network Analysis

A weighted gene co-expression network analysis (WGCNA) (v1.47) packages in R (version 4.2.3) was used [175]. For the WGCNA analysis, data was filtered, and low expression genes (FPKM < 1) were discarded to avoid misleading results. The Pearson correlation coefficients was calculated between the genes based on their expression. The threshold power was defined as 15 and the scale-free topology index was customized at 0.8, and then further analyses of hierarchical clustering, module analysis, correlation analysis between module, sample and traits, interaction relationships, and enrichment analysis were conducted.

### 3.8. Protein Interaction Network Analysis

The protein interaction network of differentially expressed genes (DEGs) were analyzed by STRING protein database. The parameter for the protein–protein interaction was set as “confidence thresholds 0.40”. The CSV file of the analyzed data was generated and imported to Cytoscape software (version 3.9.0) for visualization. The hug networks were identified using cytoHubba “Degree” [176,177].

### 3.9. Quantitative Real-Time PCR Analysis

Total RNA of the plant samples was extracted by following plant RNA extraction kit (VAZYME, Nanjing, China). The extracted RNA was used to synthesize the cDNA by utilizing a HiScript III 1St Strand cDNA synthesis kit (VAZYME, Nanjing, China) for the real-time quantitative PCR (RT-qPCR) analysis. For the RT-qPCR analysis primers were designed according to the dragon fruit CDS sequences as shown in Supplementary File S11. For each sample, three biological and three technical replicates were analyzed, and the analysis of RT-qPCR was calculated by Ct comparison (2^−ΔΔCt^) method [178].

### 3.10. Statistical Analysis

Statistical data analysis was conducted using SPSS 23.0 (SPSS Incorporation, Chicago, IL, USA) and ANOVA and Duncan tests were performed at the significance level of <0.05. The data are described as a mean ± standard deviation of three biological repeats.

## 4. Conclusions

This physiological and transcriptomic study provides valuable resources for understanding the impacts of abiotic stresses on pitaya and a unique genetic response of pitaya towards stress conditions. Notably, both the stresses of salt and copper affected pitaya growth and development. The stress effects were found to be more severe in combined treatments of salt and copper. However, an exogenous application of melatonin, in single as well as in combined stresses, enhanced pitaya growth. Salt and copper both produced high levels of oxidative stresses and decreased photosynthetic pigments. Melatonin application, in combination with stress, reversed the effects of oxidative stress and increased the content of photosynthetic pigments. Transcriptomic analysis revealed that MAPK signaling transduction, phenylpropanoid biosynthesis, plant hormonal signal transduction, photosynthesis, and pathogen interaction pathways played a key role in regulating salt and copper stress responses. Melatonin application alleviated the regulation of stress response genes in respective pathways. The candidate genes (HU06G00395, HU01G00588, HU06G00874, HU01G00989, HU11G01456, HU09G01996, HU09G00064, and HU01G00526) were associated with *GH3*, *JAZ*, *PAL*, *CCR*, *POD*, *CCoAOMT*, *PPC*, *PR1*, *ChiB*, *CAM*, and *COMT* domains and were found highly correlated with pitaya phenotypes under salt, copper, and melatonin single and combined treatments. Our findings provide insight into the essential role of melatonin in the genetic regulation of stress response pathways. Moreover, the predicted stress responsive candidate genes should be studied further, and the overexpression or knockout of these genes may be helpful in providing resistance to abiotic stresses, especially salt and copper stresses.

## Data Availability

The data will be made available on request.

## Supplementary Materials

The following supporting information can be downloaded at: https://www.mdpi.com/article/10.3390/plants13243602/s1. Supplementary File S1: Antioxidant and chlorophyll content analysis; Supplementary File S2: RNA-seq statistical analysis; Supplementary File S3: Statistics of DEGs; Supplementary File S4: Top 20 enriched GO terms in treatments; Supplementary File S5: Top 20 enriched KEGG pathways in treatments; Supplementary File S6: Differential transcriptional factors in treatments; Supplementary File S7: List of identified DEGs involved in stress responsive pathways; Supplementary File S8: Top 20 enriched GO terms in modules; Supplementary File S9: Top 20 enriched KEGG pathways in modules; Supplementary File S10: Details of identified candidate genes; Supplementary File S11: Primers designed for RT-qPCR. Supplementary Figures: Figure S1: Pre-experiment of stress treatments; Figure S2: Comparative expression analysis of common DEGs; Figure S3: Top 20 enriched GO terms in treatments; Figure S4: Top 20 enriched KEGG pathways in treatments; Figure S5: Distribution of differentially expressed transcriptional factors; Figure S6: WGCNA power value screening; Figure S7: Top 20 enriched GO terms and KEGG pathways in modules.
