# Supplementary material for: Comparative Physiological and Transcriptomics Profiling Provides Integrated Insight into Melatonin Mediated Salt and Copper Stress Tolerance in Selenicereus undatus L
Source: Plants (Basel). 2024 Dec 23;13(24):3602. doi: 10.3390/plants13243602 (PMC11678089; doi:10.3390/plants13243602)
Supplement: Supplementary file 1 [file plants-13-03602-s001.zip › plants-3364923-Supplementary figures.pdf]

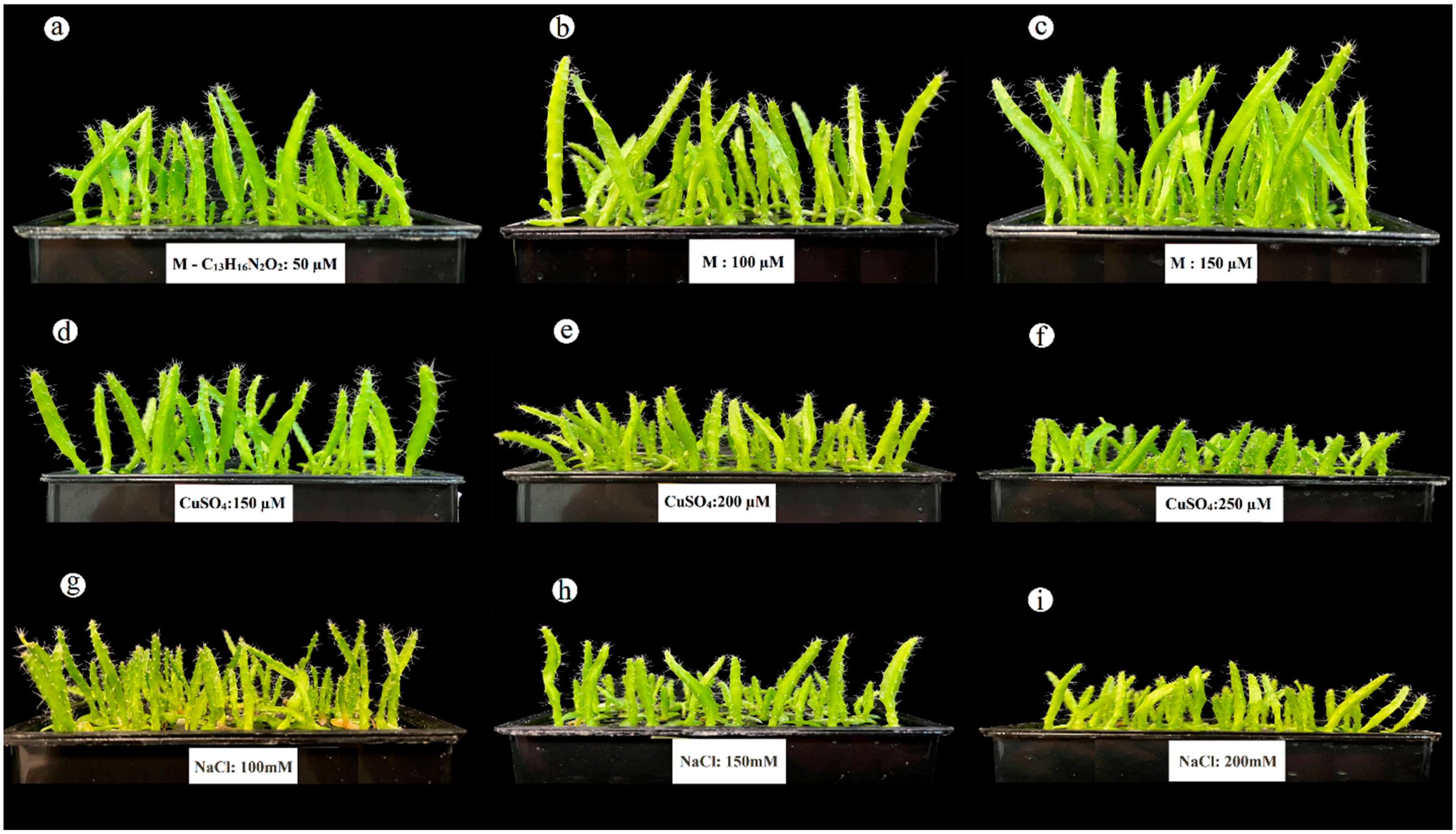

Figure S1. Pre-experiment conducted to get the optimum concentration of Melatonin (M - $C_{13}H_{16}N_2O_2$), copper (Cu – $CuSO_4$) and salt (S – NaCl). **(a)** M: 50 µM. **(b)** M: 100 µM. **(c)** M: 150 µM. **(d)** Cu: 150 µM. **(e)** Cu: 200 µM. **(f)** Cu: 250 µM. **(g)** NaCl: 100 mM. **(h)** NaCl: 150 mM. **(i)** NaCl: 200 mM.

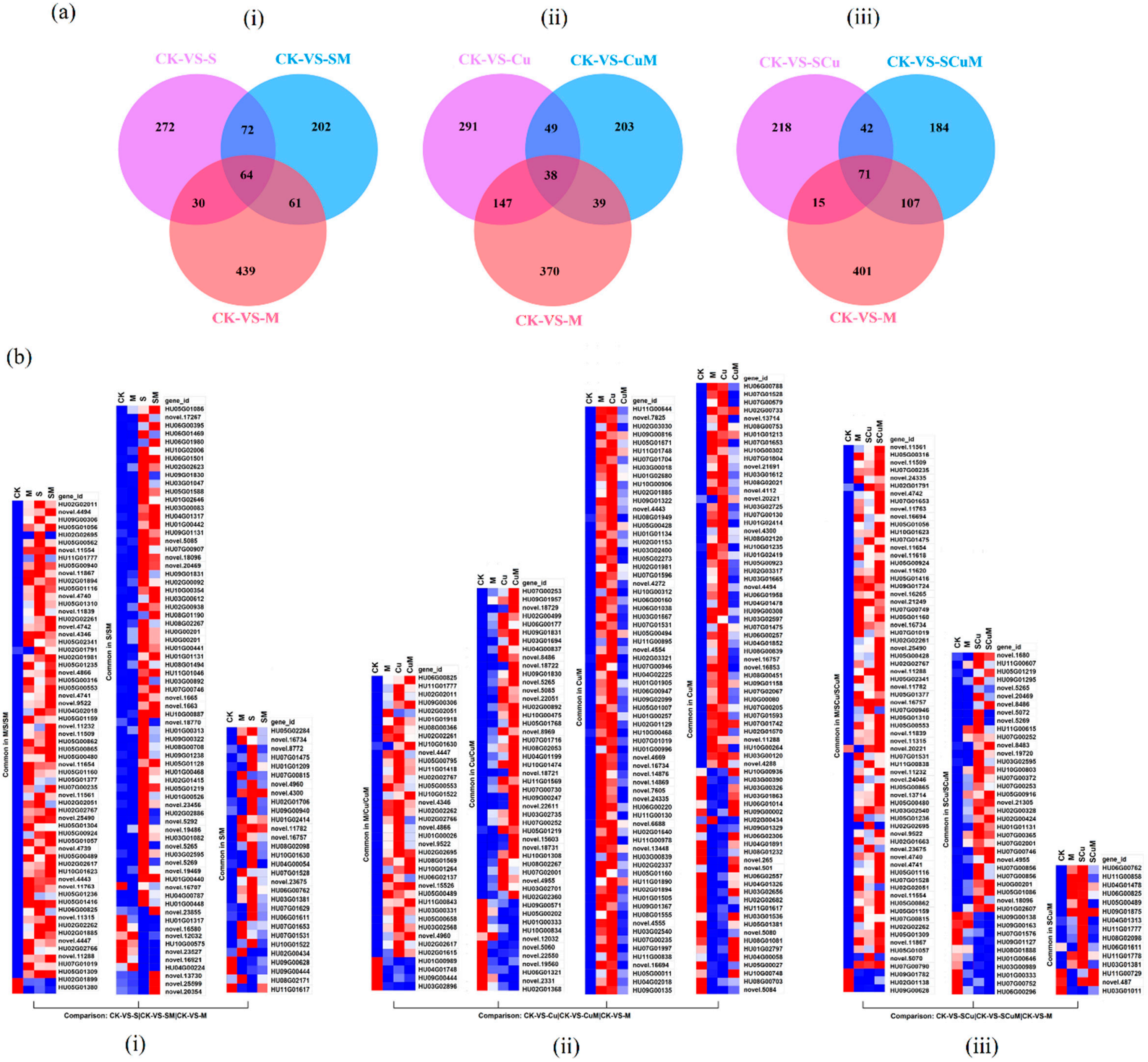

Figure S2. Comparative analysis of common DEGs identified in M, stress and stress+M treatments. **(a)** Common and unique DEGs between comparisons **(i)** S/SM/M (64 common DEGs), **(ii)** Cu/CuM/M (38 common DEGs), **(iii)** SCu/SCuM/M (71 common DEGs). **(b)** Differential expression of common DEGs **(i)** comparison: S/SM/M **(ii)** comparison: Cu/CuM/M **(ii)** comparison **(iii)** comparison: SCu/SCuM/M.

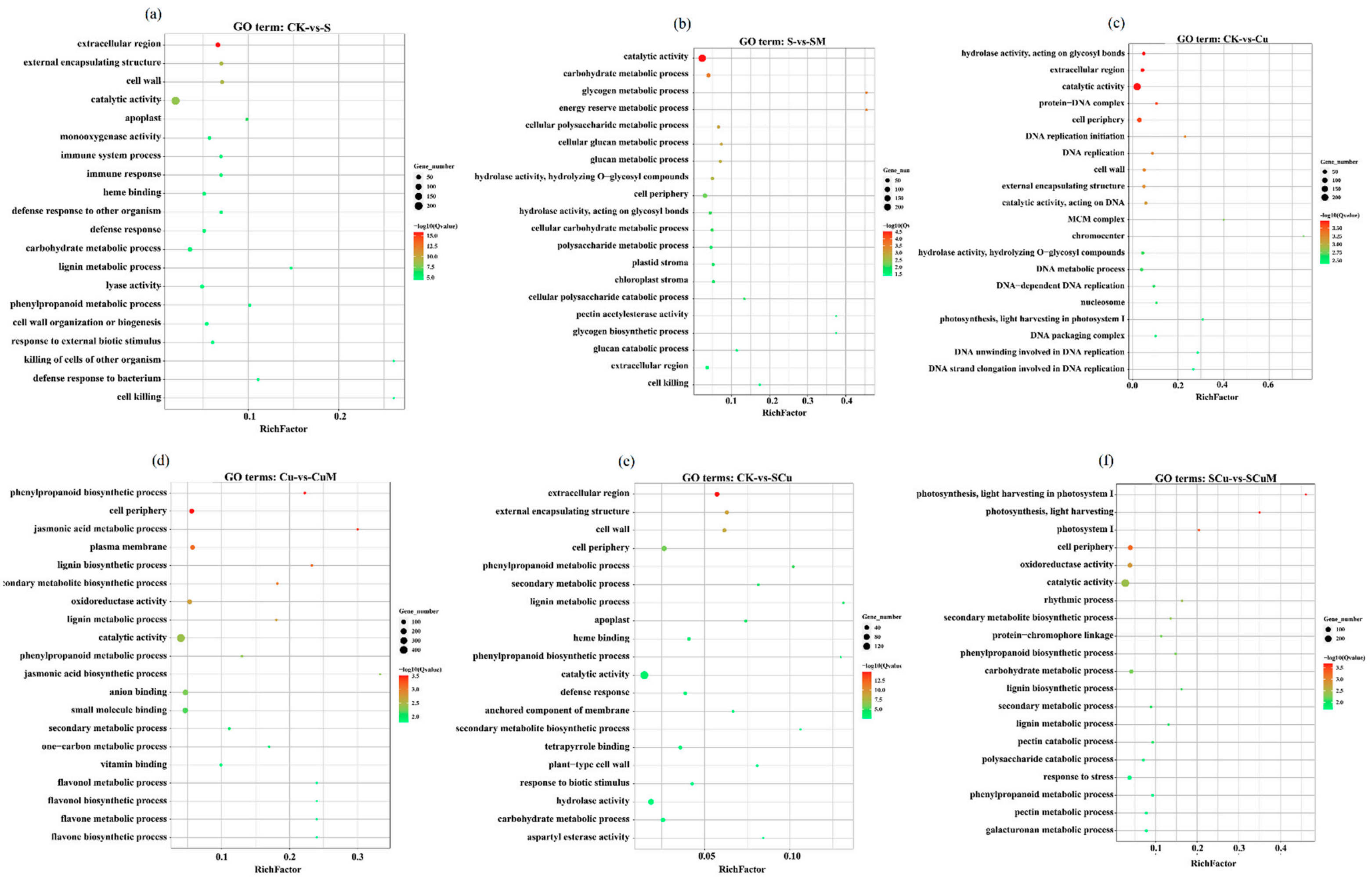

Figure S3. Top 20 GO terms of the DEGs under different treatments. **(a)** GO terms: CK-vs-S **(b)** GO terms: S-vs-SM **(c)** GO terms: CK-vs-Cu **(d)** GO terms: Cu-vs-CuM **(e)** GO terms: CK-vs-SCu **(f)** GO terms: SCu-vs-SCuM. The size of circular dots shows the number of DEGs in each GO term. The color bar from green (min) to red (max) shows the enrichment of GO terms based on Q-Values of DEGs.

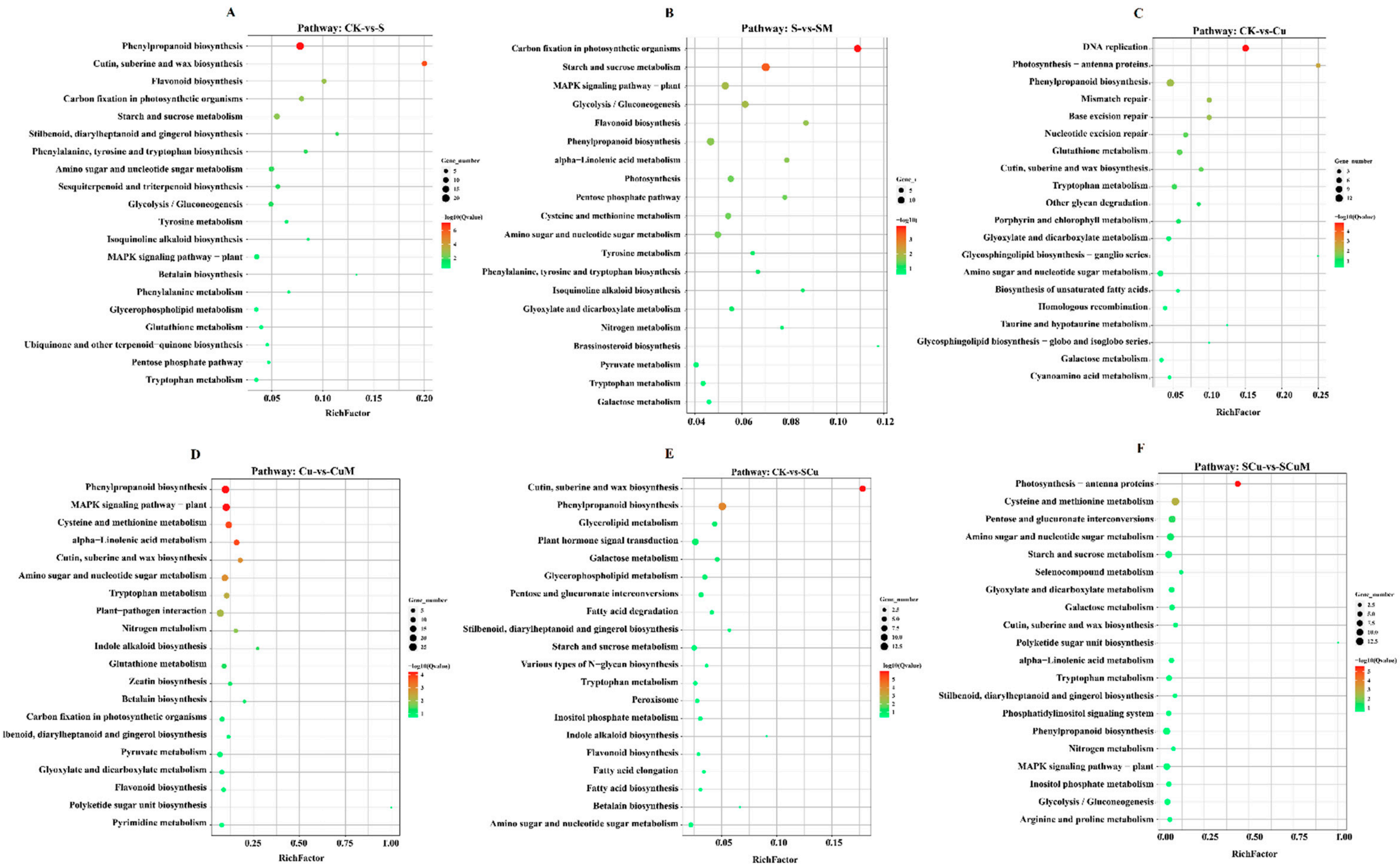

Figure S4. Top 20 KEGG enriched pathways of the DEGs under different treatments. **(A)** Pathways: CK-vs-S **(B)** Pathways: S-vs-SM **(C)** Pathways: CK-vs-Cu **(D)** Pathways: Cu-vs-CuM **(E)** Pathways: CK-vs-SCu **(F)** Pathways: SCu-vs-SCuM. The size of circular dots shows the number of DEGs in each GO term. The color bar from green (min) to red (max) shows the enrichment of GO terms based on Q-Values of DEGs.

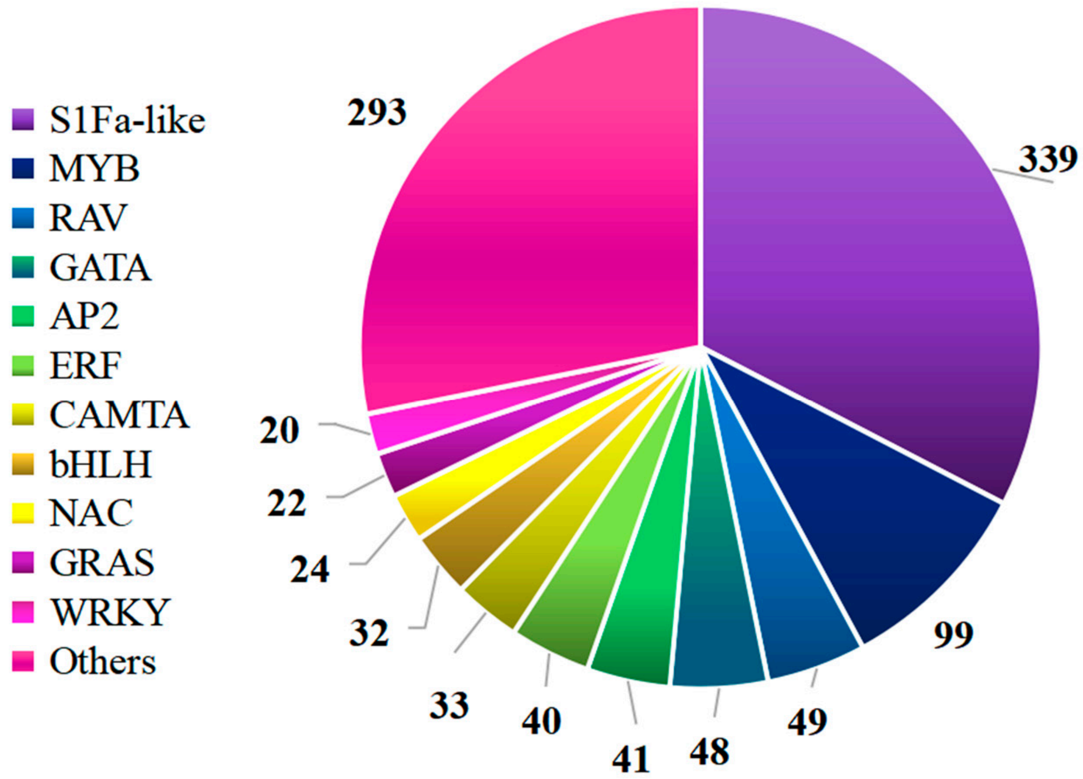

Figure S5. Distribution of transcription factor in differentially expressed pitaya (*Selenicereus undatus*) transcriptome sequences. Pie plot exhibit that *S1Fa-like, MYB*, *AP2/ARF, RAV, GATA, CAMTA, bHLH*, *NAC, GRAS* and *WRKY* are the largest transcription factors families expressed in pitaya under different treatments of salt, copper and melatonin.

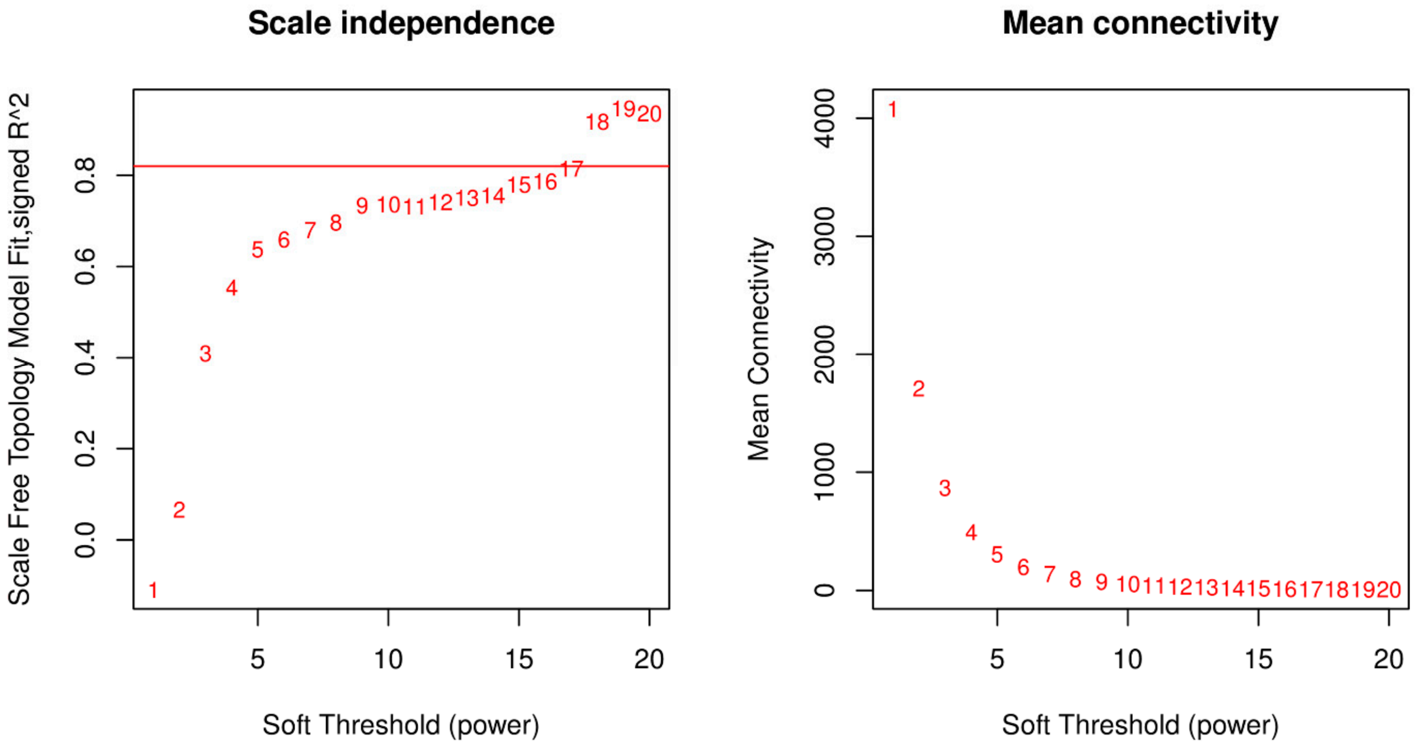

Figure S6: Relationship between the soft-thresholding power and the scale-free topology model fit (R^2) and the effect of soft-thresholding power on mean connectivity (average degree of nodes in the network).

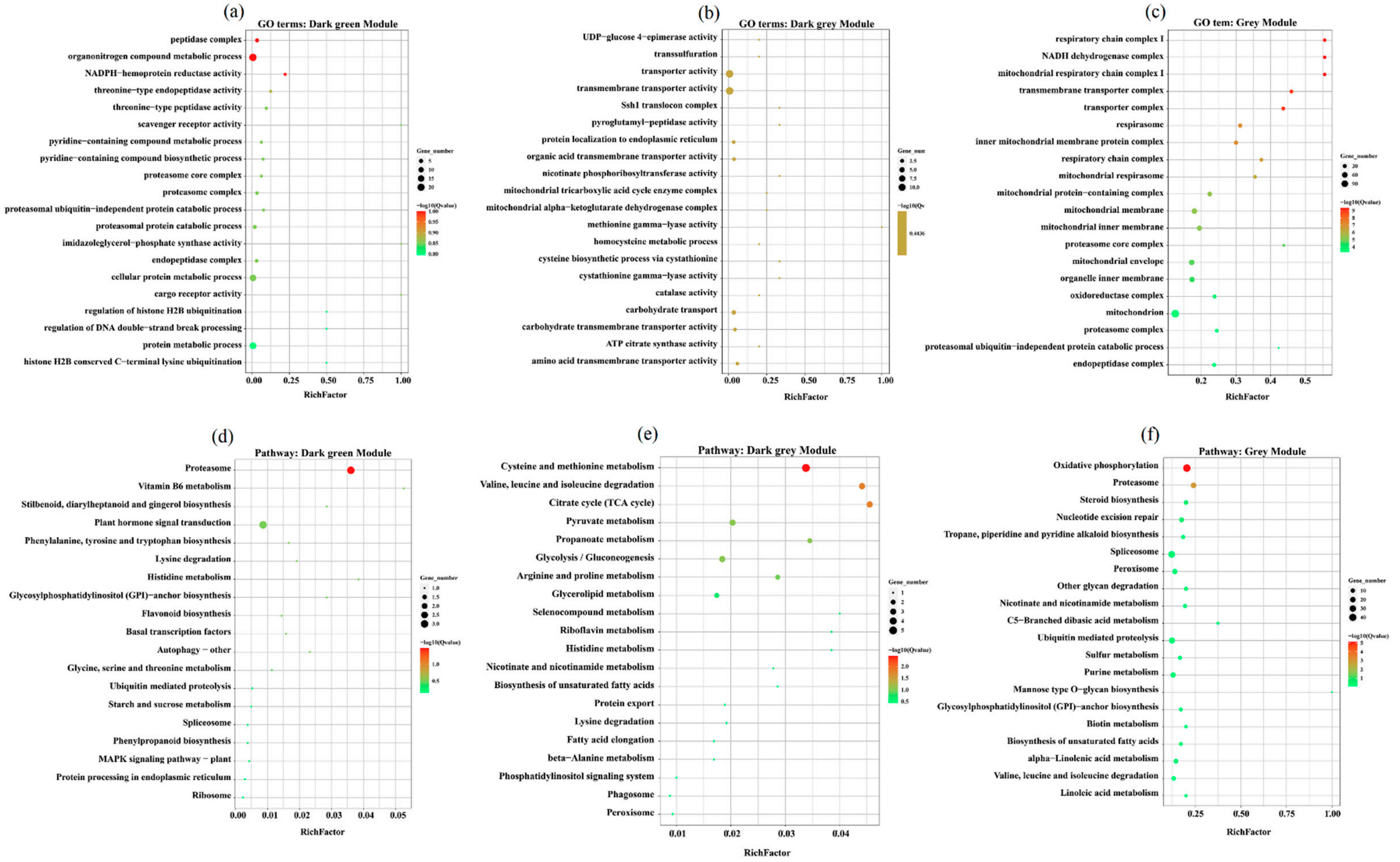

Figure S7. Top 20 GO and KEGG enriched pathways of the DEGs in different modules **(a)** GO terms: Dark green module **(b)** GO terms: Dark grey module **(c)** GO terms: Grey module **(d)** Pathways: Dark green module **(e)** Pathways: Dark grey module **(f)** Pathways: Grey module. The size of circular dots shows the number of DEGs in each GO term. The color bar from green (min) to red (max) shows the enrichment of GO terms based on Q-Values of DEGs.
